# BCR::ABL1 Tyrosine Kinase Inhibitors During Pregnancy, a Disproportionality Analysis of Vigibase

**DOI:** 10.1002/cpt.3730

**Published:** 2025-05-29

**Authors:** Aurélie Cabannes‐Hamy, Rayan Kabirian, Floriane Jochum, Kevin Bihan, Elise Dumas, Florence Coussy, Enora Laas, Philippe Rousselot, Sylvain Choquet, Madalina Uzunov, Laurent Salomon, Fabien Reyal, Anne‐Sophie Hamy, Paul Gougis

**Affiliations:** ^1^ Hematology Centre Hospitalier de Versailles Site André Mignot Le Chesnay‐Rocquencourt France; ^2^ Residual Tumor & Response to Treatment Laboratory RT2Lab, INSERM, U932 Immunity and Cancer, Institut Curie, Université Paris Cité Paris France; ^3^ Department of Gynecology Strasbourg University Hospital Strasbourg France; ^4^ Department of Pharmacology, Institut National de la Santé et de la Recherche Médicale (INSERM), Assistance Publique – Hôpitaux de Paris (AP‐HP), Clinical Investigation Center (CIC‐1901), Sorbonne Université Pitié‐Salpêtrière Hospital Paris France; ^5^ Paris Pitié‐St Antoine Regional Pharmacovigilance Center, Medical Pharmacology Department APHP Sorbonne University Hospital Group Paris France; ^6^ Department of Mathematics École Polytechnique Fédérale de Lausanne Lausanne Switzerland; ^7^ Department of Medical Oncology Institut Curie, Université Paris Cité Paris France; ^8^ Department of Breast, Gynecological and Reconstructive Surgery Institut Curie, Université Paris Cité Paris France; ^9^ University of Versailles Paris‐Saclay Versailles France; ^10^ Department of Hematology Assistance Publique – Hôpitaux de Paris (AP‐HP), Sorbonne Université, Pitié‐Salpêtrière Hospital Paris France; ^11^ Department of Obstetrics, Surgery, Fetal Medicine and Imaging Hôpital Necker‐Enfants Malades Paris France; ^12^ Department of Surgical Oncology Institut Godinot Reims France; ^13^ Department of Medical Oncology, Sorbonne Université Assistance Publique – Hôpitaux de Paris (AP‐HP), Pitié‐Salpêtrière Hospital, Institut Universitaire de Cancérologie Paris France

## Abstract

Tyrosine kinase inhibitors (TKIs) targeting BCR::ABL1 have greatly improved the survival of patients with chronic myeloid leukemia (CML), and their teratogenicity appears as an important factor for individuals of childbearing potential. This study aims to investigate pregnancy and fetal/newborn adverse outcomes resulting from exposure to BCR::ABL1‐TKIs during pregnancy. For this disproportionality analysis, we used the WHO's global VigiBase up to January 2024, and included reports involving pregnancy, antineoplastic treatment during pregnancy, and cancer. The exposure group consisted of reports mentioning BCR::ABL1‐TKIs at any time during pregnancy. The primary outcome was the reporting odds ratio (ROR) of maternal‐fetal complications in the BCR::ABL1‐TKIs group compared to other anticancer treatments. The analysis included 3,389 reports (TKI = 969; other = 2,420). In the BCR::ABL1‐TKI‐exposed group, the mean age was 28.9 years, and 724 patients (92.2%) were treated for CML. BCR::ABL1‐TKIs were mainly imatinib (*n* = 642, 66.3%), nilotinib (*n* = 218, 22.5%), and dasatinib (*n* = 127, 13.1%) reported without other non‐TKI anticancer agents(92.3%). Compared to other anticancer drugs, overreported outcomes with TKIs included hydrops fetalis (ROR = 13 [95%CI = 1.5–110], *P* = 0.009), polyhydramnios (ROR = 5 [1.3–20], *P* = 0.02), and threatened preterm labor (ROR = 10 [1.1–90], *P* = 0.03). When analyzing specific molecule effects, hydrops fetalis (ROR = 27 [5.4–130], *P* = 0.001) and polyhydramnios (ROR = 13 [3.3–54], *P* = 0.004) were overreported with dasatinib. In this large cohort of 969 individuals exposed to TKIs during pregnancy, dasatinib use was associated with most BCR::ABL1‐TKI‐specific toxicities and should be avoided during pregnancy. Fewer or no adverse events were overreported with imatinib and nilotinib.

**Study Highlights**

**WHAT IS THE CURRENT KNOWLEDGE ON THE TOPIC?**

BCR::ALB1 Tyrosine Kinase Inhibitors, have been historically associated with pregnancy and newborn complications. This study analyzes the largest cohort of maternofetal exposure to BCR::ABL1‐targeting TKIs, assessing pregnancy and neonatal outcomes.

**WHAT QUESTION DID THIS STUDY ADDRESS?**

Limited data on the safety of TKIs during pregnancy are available, necessitating a comprehensive evaluation of potential risks.

**WHAT DOES THIS STUDY ADD TO OUR KNOWLEDGE?**

This study identifies an increased risk of hydrops fetalis, polyhydramnios, and other pregnancy complications after BCR::ALB1 TKIs exposure. Imatinib and nilotinib were associated with fewer fetal and obstetric toxicities than dasatinib.

**HOW MIGHT THIS CHANGE CLINICAL PHARMACOLOGY OR TRANSLATIONAL SCIENCE?**

This study provides insights that could influence treatment decisions during pregnancy, suggesting cautious use of certain TKIs such as dasatinib and prompting further research into safer options for pregnant patients.

Chronic myeloid leukemia (CML) is a myeloproliferative neoplasm driven by the fusion gene *BCR::ABL1*, which encodes a constitutively active and targetable tyrosine kinase.

Since the early 2000s, the introduction of BCR::ABL1 tyrosine kinase inhibitors (TKIs) has dramatically improved the outcome of patients with CML and is now the cornerstone of CML management. Consequently, patients with CML now have a life expectancy comparable to the general population, aligning their outlook with non‐leukemic peers. With prognosis improvement, objectives have thus moved to treatment‐free remission, quality of life, and conception: pregnancy has emerged as an important aspect to be considered in patients of childbearing potential. CML accounts for up to 10% of pregnancy‐associated leukemias, with an annual incidence of 1 per 100,000 pregnancies.^1^, ^2^ Also, the median age of onset is younger – about 30 to 40 years old– in emerging nations, compared to Western countries,^3^, ^4^, ^5^ where imatinib, dasatinib, and nilotinib are more and more widely prescribed since they are available as generics. This growing use of TKIs in younger patients of reproductive age has raised concerns about their safety during pregnancy, as data on maternal‐fetal outcomes from in utero exposure remain limited.

In a study of 125 pregnant women taking imatinib, 50% had normal deliveries, but 12 infants (9.6%) were born with congenital abnormalities, including 2 with notably similar complex malformations including exomphalos, renal agenesis, and hemivertebrae, raising significant concerns.^6^ Regarding dasatinib, a study exploring the BMS pharmacovigilance database^7^ identified 46 women with known pregnancy outcomes following exposure to the drug. Maternal complications included intrauterine growth restriction and premature birth, while congenital abnormalities included one bilateral pyelocaliceal dilatation, two hydrops fetalis associated with central nervous system abnormalities (one premature closure of cranial vault sutures and one microretrognathia associated to hypertelorism). Data regarding other BCR::ABL1 TKIs remain limited to clinical case report series.^8^, ^9^, ^10^, ^11^, ^12^, ^13^ However, to date, no comparative study has been done, and there is a need to better characterize the risk of maternofetal adverse outcomes with these agents.

The World Health Organization VigiBase, a global pharmacovigilance database, contains over 36 million individual case safety reports of adverse drug reactions to date from more than 130 countries since 1967. This database is a valuable resource in uncovering new adverse drug reactions.^14^ In onco‐hematology, good pharmacovigilance practice guidelines^15^ stipulate that medical professionals should closely observe and report any instances of pregnancy both during clinical trials and regular treatment. Utilizing VigiBase, earlier investigations revealed that exposure to anti‐HER2 treatments during pregnancy was linked to a significant occurrence of oligohydramnios, probably due to kidney issues in fetuses or newborns. In very rare situations, this condition led to pulmonary hypoplasia.^16^ Another study on immune‐checkpoint inhibitors found no frequent toxicity pattern using this class of drugs.^17^

The main objective of this study was to perform a disproportionality analysis of pregnancy and fetal or newborn adverse outcomes after exposure to BCR::ABL1 TKI compared to exposure to other anticancer drugs. Secondary analysis included the investigation of molecule‐specific toxicities.

## METHODS

### Study design

This cohort study utilizes pharmacovigilance individual case safety reports from VigiBase, the WHO global database of reported potential side effects of medicinal products, which is developed and maintained by the Uppsala Monitoring Centre. We performed a case/non‐case disproportionality analysis to evaluate the association between maternal and fetal/newborn adverse outcomes and exposure to TKI compared to exposure to other anticancer agents within reports of patients with cancer and anticancer drug exposure during pregnancy.

### Data query and report extraction

VigiBase was queried on January 1, 2024, with Medical Dictionary for Regulatory Activities (MedDRA) version 26.1. We extracted VigiMatch deduplicated reports from VigiBase containing one or more pregnancy‐related reactions and suspecting one or more anticancer drugs.

- “Reactions” related to pregnancy were defined as any reaction with a reported term falling in the following MedDRA dictionary categories: Pregnancy, puerperium and perinatal conditions (system organ classification—SOC)/fetal and neonatal investigations (high‐level group term—HLGT)/neonatal and perinatal conditions (HLGT)/neonatal respiratory disorders (HLGT)/exposures associated with pregnancy, delivery and lactation (high‐level term—HLT)/fetal therapeutic procedures (HLT)/induced abortions (HLT)/obstetric therapeutic procedures (HLT). Details are summarized in **Table** **S1**.
- A report was suspecting an anticancer drug when one or more anticancers were notified as “Suspect” (or “Interacting”). Anticancer drugs were any drugs from the “antineoplastic” Anatomical Therapeutic Classification (ATC) group L01.

### Data cleaning and exclusion criteria

We ensured that only reports mentioning pregnancy‐associated conditions/exposure were retained by discarding reports with terms secondarily associated with pregnancy. Only reports with terms primarily associated with pregnancy as a main SOC, HLGT, or HLT were retained.

Reports were then analyzed to discard:

- Reports with no mention of a cancer diagnosis or with an antineoplastic drug from the L01 ATC group prescribed for a non‐cancer indication (*e.g*., prescription of methotrexate for rheumatoid arthritis or of alemtuzumab for multiple sclerosis);
- Reports with drug mapping problems or adverse event mapping problems (**Table** **S2**).
- Dyads, that is, maternal and fetal reports referring to the same case, were detected using an in‐house information‐entropy based algorithm and merged (cf. **Supplemental Methods**).

### Modality of exposure during pregnancy

Modality of anticancer drug exposure were identified using preferred terms notified in reports (**Table** **S3**, terms and modality of exposure). Exposure types were: exposure before pregnancy, exposure during pregnancy, exposure via breast milk, exposure via semen, and exposure via skin. The exposure’ timing by trimester was noted when available. Reports with a biological sex notified as male were considered to be exposed via semen. Reports with notification of a term associated with exposure via skin or semen were excluded. Reports with exposure via breast milk or before pregnancy and no specific mention of exposure during pregnancy were also discarded. All other reports were considered to have exposure to anticancer drugs during pregnancy.

### Definition of exposure groups

We considered the following drugs to be TKI targeting BCR::ABL1: imatinib, nilotinib, dasatinib, ponatinib, bosutinib, asciminib and radotinib. Any report from the study analysis with a mention of a BCR::ABL1 TKI drug was qualified as a “TKI exposure group”. Each report within the TKI exposure group was individually analyzed. Reports with other anticancers and no mention of BCR::ABL1 TKI were qualified as the “exposure to other anticancers” group.

### Definition of cases and non‐cases for maternal and fetal/newborn outcomes

In this disproportionality analysis, cases were reported with mention of a maternal and fetal/newborn adverse events categorized from MedDRA preferred terms in VigiBase. They constituted 45 individual maternal‐fetal adverse outcomes regrouped into seven categories for the purposes of this study: Abortion (induced and spontaneous); Stillbirth/fetal death; Congenital malformation; Pregnancy complication; Preterm birth; Neonatal complication; and Delivery complication. A detail of fetal toxicity subtypes explored is available in **Table** **S4**. Some adverse outcomes were deemed not clinically relevant and were discarded (**Table** **S5**).

For the independent analysis of each adverse event, cases were defined as study population reports with a mention of the adverse event. Non‐cases were defined as all other study reports with no mention of the adverse event.

### Statistical analysis

We performed a case/non‐case study using a disproportionality analysis to evaluate the association between an adverse outcome of interest and an exposure. The reporting odds ratio (ROR) was defined as the ratio of the odds of the adverse pregnancy or fetal/newborn outcome of interest with exposure to TKI to the odds with exposure to other anticancer drugs. The signal was adjusted for multiple testing based on the number of molecules investigated with a number of occurrences of 10 or more in the whole cohort.

A signal was considered to be present when statistically significant disproportionality, with the lower end of the ROR confidence interval, ROR_025_, was over 1 and the number of occurrences was 2 or more. The study population is described in terms of frequencies for qualitative variables or medians and interquartile ranges [IQR] for quantitative variables. Associations between categorical variables were assessed with Fisher's tests. *P*‐values of less than 0.05 were considered statistically significant.

### Mitigation of biases and confounding factors

To assess the robustness of our results, we conducted sensitivity/subgroup analyses on reports for which a single class of treatment was used (BCR::ABL1 TKI in the TKI‐exposed group). For this analysis, any report with a combination of drug classes, such as cytotoxic+TKI, was discarded. To identify specific effects of each TKI, we conducted a disproportionality analysis for each TKI molecule independently with substantial numbers (*N* > 10): imatinib, nilotinib, dasatinib, and ponatinib. Another sensitivity analysis was done within the population of reports for which a chronic myeloid leukemia was identified. To assess the impact of CML on our results, we also evaluated the impact of BCR::ABL1 TKIs excluding reports with a diagnosis of CML.

To limit the impact of biases, we also identified confounding variables using a Directed Acyclic Graph (**Figure** **S1**). The “year of report”, the “country of report”, “patient's age” and the “cancer type” were main variables that needed adjustment to limit confounding factors. The odds ratio for the risk of each toxicity was then evaluated using a multivariate analysis by logistic regression with adjustment on these variables. Missing data were grouped within a single level of value for each variable.

### Data protection

The data in the VigiBase database are anonymized, and it is not possible to access personal information about the patients or the individuals reporting the cases. We adhered to all applicable legislation such as, but not limited to, EU and national legislation regarding the protection of personal data (e.g., the Data Protection Directive 95/46/ EC and Regulation (EC) No. 45/2001, as applicable). The study follows the Reporting of a Disproportionality Analysis for Drug Safety Signal Detection Using Individual Case Safety Reports in PharmacoVigilance (READUS‐PV) checklist (**Table** **S6**) and the Strengthening the Reporting of Observational Studies in Epidemiology (STROBE) reporting guideline for case–control studies (**Table** **S7**).^18^ The institutional review board of Institut Curie (Comité de Revue Institutionnelle‐CRI Data) granted study approval. The data used in this study are available upon request from the corresponding author. The statistical analyses were performed using RStudio Version 2023.12.1 + 402.

## RESULTS

### Reports characteristics

**Figure 1:**
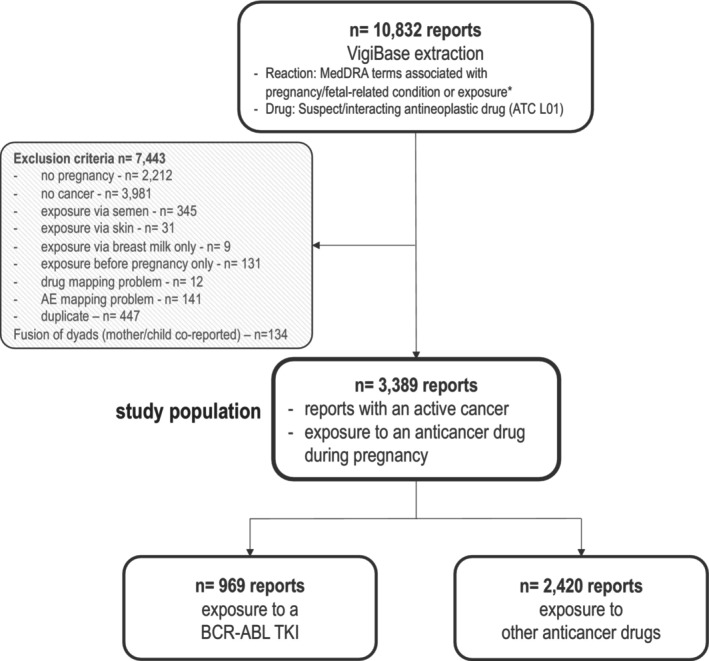
Study flow‐chart. All reports were extracted from VigiBase using Vigilyze. Details of data extraction are provided in **Table** **S1**. Terms associated secondarily with this request were not specific to pregnancy and were discarded. Reports also mentioned a suspect or interacting drug from the anatomical and therapeutic classification L ATC L01 class (antineoplastic drugs), which could have been prescribed for a cancer or a non‐cancer indication. *MedDRA terms queried for “Reaction” were: Pregnancy, puerperium, and perinatal conditions (SOC), Fetal and neonatal investigations (HLGT), Neonatal and perinatal conditions (HLGT), Neonatal respiratory disorders (HLGT), Exposures associated with pregnancy, delivery, and lactation (HLT), Fetal therapeutic procedures (HLT), Induced abortions (HLT), Obstetric therapeutic procedures (HLT). ATC, Anatomical Therapeutic Chemical classification; MedDRA, Medical Dictionary for Regulatory Activities; TKI, Tyrosine Kinase Inhibitor.

We extracted 10,832 deduplicated reports and retained 3,389 reports of pregnant individuals exposed to anticancer drugs for the final analysis (**Figure** **1**) (TKI exposure, *n* = 969; other anticancer drugs, *n* = 2,420). In the group exposed to TKI, most reports were from Asia (*n* = 402, 41.5%), and the mean (SD) age was 28.9(9.5) years old. Within the TKI group, chronic myeloid leukemia (*n* = 724, 92.2%) was the most frequent cancer, and other malignancies included acute leukemia (*n* = 24, 3.1%), non‐otherwise specified/other leukemia (*n* = 21, 2.7%) and sarcoma (*n* = 16, 2.0%). In the non‐exposed group, breast cancer (*n* = 918, 44.3%), other solid tumors (*n* = 409, 19.7%) and lymphoma (*n* = 377, 18.2%) were the most frequent types of cancer (**Table** **1**; **Figure** **S2**).

**Table 1:** Reports characteristics from the cohort with a report of a BCR::ABL1 tyrosine kinase inhibitors (TKI) or with other anticancer drugs

Variable name	Level	Overall, *n* (%)	BCR::ABL1 TKIs, *n* (%)	Other anticancer drugs, *n* (%)	*P*
		3,389	969 (28.6)	2,420 (71.4)	
Age (years)		28.5 (12.6)	28.9 (9.5)	28.3 (14.0)	0.42
Country group	Asia	571 (16.8)	402 (41.5)	169 (7.0)	<0.001
	USA	1,555 (45.9)	246 (25.4)	1,309 (54.1)	
	Other country	1,263 (37.3)	321 (33.1)	942 (38.9)	
Notifier type	Consumer or non‐health professional	321 (10.1)	160 (17.4)	161 (7.1)	<0.001
	Physician or pharmacist	1,647 (51.8)	562 (61.2)	1,085 (48.0)	
	Other health professional	1,210 (38.1)	197 (21.4)	1,013 (44.8)	
Number of notifiers	1	2,858 (89.9)	691 (75.2)	2,167 (95.9)	<0.001
	2+	320 (10.1)	228 (24.8)	92 (4.1)	
Year of first report	2009 or before	289 (8.5)	80 (8.3)	209 (8.6)	<0.001
	2010–2014	778 (23.0)	143 (14.8)	635 (26.2)	
	2015–2019	1,357 (40.0)	479 (49.4)	878 (36.3)	
	2020–2022	965 (28.5)	267 (27.6)	698 (28.8)	
Number of suspect/interacting drugs	1	1,460 (43.1)	746 (77.0)	714 (29.5)	<0.001
	2	595 (17.6)	126 (13.0)	469 (19.4)	
	3+	1,334 (39.4)	97 (10.0)	1,237 (51.1)	
Clinical trial	Clinical trial	54 (1.6)	10 (1.0)	44 (1.8)	0.13
	Routine care	3,335 (98.4)	959 (99.0)	2,376 (98.2)	
Cancer type	Chronic myeloid leukemia	727 (25.5)	724 (92.2)	3 (0.1)	<0.001
	Acute leukemia	214 (7.5)	24 (3.1)	190 (9.2)	<0.001
	Breast	918 (32.1)	0 (0.0)	918 (44.3)	<0.001
	Lymphoma	377 (13.2)	0 (0.0)	377 (18.2)	<0.001
	Solid tumor other or NOS	409 (14.3)	0 (0.0)	409 (19.7)	<0.001
	Sarcoma	98 (3.4)	16 (2.0)	82 (4.0)	<0.001
	Hematological other or NOS	90 (3.2)	3 (0.4)	87 (4.2)	<0.001
	Leukemia other or NOS	38 (1.3)	21 (2.7)	17 (0.8)	0.23
	Digestive other or NOS	21 (0.7)	2 (0.3)	19 (0.9)	<0.001
Suspect molecule class	Cytotoxic	1925 (56.8)	43 (4.4)	1882 (77.8)	<0.001
	Molecular targeted therapy	1816 (53.6)	969 (100.0)	847 (35.0)	<0.001
	Immunotherapy	154 (4.5)	32 (3.3)	122 (5.0)	0.035
	Endocrine therapy	71 (2.1)	0 (0.0)	71 (2.9)	<0.001
	Other or NOS anticancer	36 (1.1)	1 (0.1)	35 (1.4)	0.001
	Comedication	931 (27.5)	138 (14.2)	793 (32.8)	<0.001

USA, United States of America; NOS, not otherwise specified.

Quantitative values are represented as mean and standard deviation. Patients could have two cancers simultaneously.

Missing data.

For BCR::ABL1 TKIs: age (years), *n* = 459; notifier type, *n* = 50; number of notifiers, *n* = 50; number of cancers, *n* = 184.

For other anticancer drugs: age (years), *n* = 1,431; notifier type, *n* = 161; number of notifiers, *n* = 161; number of cancers, *n* = 349.

### Exposure to anticancer drugs

The molecules involved in TKI cases (*n* = 969) were imatinib (*n* = 642, 66.3%), nilotinib (*n* = 218, 22.5%), dasatinib (*n* = 127, 13.1%), ponatinib (*n* = 10, 1.0%), bosutinib (*n* = 4, 0.4%), radotinib (*n* = 2, 0.2%) and asciminib (*n* = 1, 0.1%). Among them, 27 patients received two TKIs, and 4 received three. TKIs were used in combination with non‐TKI anticancer agents in 73 participants (7.5%): 40 (4.1%) with cytotoxic chemotherapy and 33 (3.4%) with interferon‐alfa combinations (**Figure** **S3**). In the other anticancer group, 1,882 (77.8%) reports mentioned a cytotoxic agent, and 847 (35.0%) another molecular targeted therapy mainly (**Table** **1**).

### Pregnancy and/or fetal or newborn outcomes

**Figure 2:**
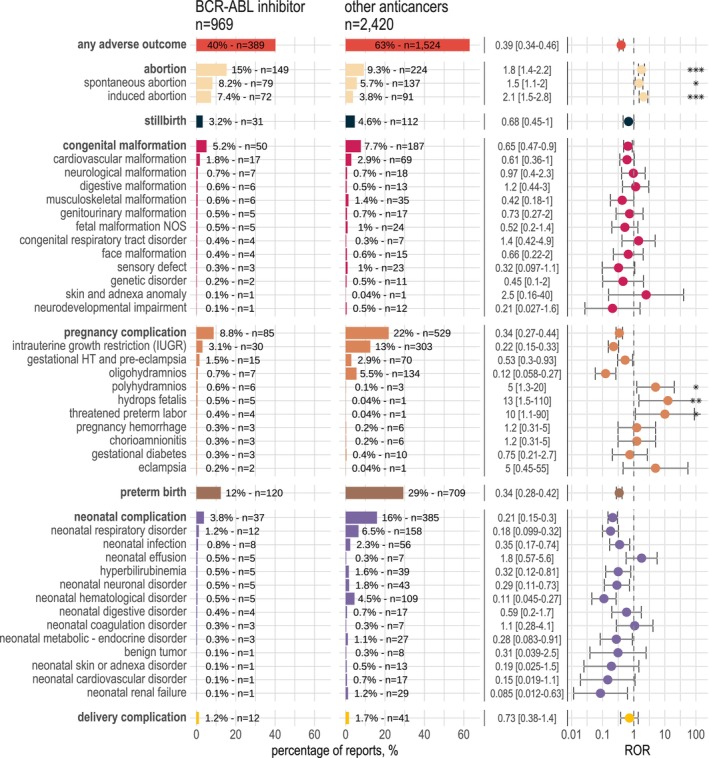
Description and reporting odds ratio (ROR) of pregnancy and fetal/newborn adverse outcomes with exposure to BCR::ABL1 tyrosine kinase inhibitors (TKI) compared to exposure to other anticancer drugs. Bars represent the percentage of each outcome divided by the total number of reports. Counts are annotated. RORs are displayed as logarithmic data for the purpose of data visualization. Among the 45 pre‐specified maternal‐fetal adverse outcome types, we represented (**Table** **S4**) toxicities for which at least one case was found in the TKI‐exposed group. *P*‐values: *P‐value ≤0.05, **P‐value ≤0.01, ***P‐value ≤0.001. When the *N* observed was one, the result was not considered significant. HT, Hypertension; NOS, Not Otherwise Specified; TKI, Tyrosine Kinase Inhibitor.

Pregnancy or fetal/newborn adverse outcomes were identified in 389 reports (40.1%) in the TKI group and 1,524 reports (63.0%) in the other anticancers group, ROR = 0.39 [95%CI = 0.34–0.46] (**Figure** **2**, **Table** **S8**). When used as a single class, 40.1% had an adverse outcome (*n* = 359/896), and 64.4% (*n* = 47/73) when TKIs were combined with other anticancer agents (**Figure** **S4**). The 5 complications most frequently reported in the TKI group were abortion (*n* = 149,15%), of which 79 were spontaneous (8.2%) and 72 induced (7.4%), preterm birth (*n* = 120,12%), stillbirth (*n* = 31,3.2%), intrauterine growth restriction (*n* = 30,3.1%) and cardiovascular malformation (*n* = 17,1.8%).

The ROR was significantly higher than 1 in TKI‐exposed reports for hydrops fetalis (ROR = 13 [95%CI = 1.5–110], *P* = 0.009), polyhydramnios (ROR = 5 [95%CI = 1.3–20], *P* = 0.02), abortion (ROR = 1.8 [95%CI = 1.4–2.2], *P* = 6 × 10^−7^) and threatened preterm labor (ROR = 10 [95%CI = 1.1–90], *P* = 0.026) (**Figure** **2**). Threatened preterm labor was an isolated complication, and effusions (hydrops fetalis, polyhydramnios, neonatal effusion) could be reported alone or together (**Figure** **S5**).

**Figure 3:**
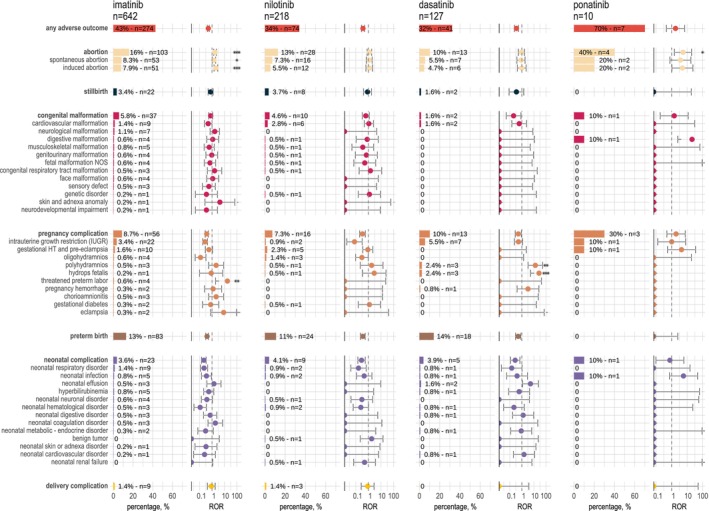
Description and reporting odds ratio (ROR) of pregnancy and fetal/newborn adverse outcomes with exposure to BCR::ABL1 tyrosine kinase inhibitors (TKI) individually. Bars represent the percentage of each outcome divided by the total number of reports. Counts are annotated. RORs are displayed as logarithmic data for the purpose of data visualization. *P*‐values: *P‐value ≤0.05, **P‐value ≤0.01, ***P‐value ≤0.001. When the *N* observed was one, the result was not considered significant. HT, Hypertension; NOS, Not Otherwise Specified.

In subgroups analysis by molecules, hydrops fetalis (ROR = 26 [95%CI = 5.3–130], *P* = 0.001) and polyhydramnios (ROR = 13 [95%CI = 3.2–53], *P* = 0.004) were significantly overreported with dasatinib exposure compared to other anticancers. In reports that mentioned exposure to imatinib, the ROR was significantly higher than 1 for threatened preterm labor (ROR = 17 [95%CI = 1.9–154], *P* = 0.005) and abortion (ROR = 1.8 [95% CI = 1.4–2.2], *P* = 1 × 10^−5^) (**Figure** **3**, **Table** **S9**). For ponatinib exposure, abortion (ROR = 5.4 [95%CI = 1.5–19], *P* = 0.02) was also overreported. No significant overreporting in any pregnancy and fetal/newborn adverse outcome was observed with nilotinib exposure.

### Sensitivity and multivariable analysis

In the multivariate analysis (**Figure** **S6**), after adjustment for the year of first report in Vigibase and country of the reports, individual's age, and tumor type, main toxicity signals remained with an odds ratio (OR) significantly higher than 1 for abortion (OR = 1.60 [95%CI = 1.2–2.1], *P* = 3 × 10^−3^) and for polyhydramnios (OR = 12.6 [95%CI = 1.8–110], *P* = 0.02).

In the sensitivity analysis performed on the subpopulation of patients receiving only anticancer treatment in monotherapy (*n* = 1,697, with 896 receiving TKI in monotherapy and 801 other anticancers) trends toward increased risk of polyhydramnios, hydrops fetalis, and threatened preterm labor remained (**Figures** **S4, S7**).

Sensitivity analysis performed on the subpopulation of CML by types of TKI molecules identified similar adverse drug reaction (ADR) outcomes among patients receiving dasatinib with a significant overreporting of hydrops (ROR = 8.7 [1.4–53]) and polyhydramnios (ROR = 8.7 [1.4–53]) (**Figure** **S8**). In the subpopulation of 43 reports with no CML, abortion rates were similar in the BCR::ABL1 TKI‐exposed group compared to other anticancers (ROR = 1.1 [0.38–3.0]) (**Figure** **S9**).

### Reports of interest

The overall number of cases of congenital malformation/pregnancy complication is small. However, patterns were observed that may indicate potential safety concerns. Within the whole cohort, 5 reports included a mention of hydrops fetalis and BCR::ABL1 TKI exposure: one was associated with imatinib, three with dasatinib, and one with nilotinib (**Table** **2**). Three of these also reported neonatal death. Moreover, 3 other reports were related to effusions and possibly related to hydrops fetalis, although not reported as such: one report of fetal ascites, skin edema, pericardial effusion leading to neonatal death with imatinib during the first trimester (T1) (case iv), one fetal pleural effusion with nilotinib at T1 (case xiii) and one fetal ascites with induced abortion after short‐term imatinib exposure (case vii). Among these, three reports mentioned atrial septal defects together with hydrops fetalis with nilotinib (case xiii) or imatinib (cases iii, iv).

**Table 2:** Reports of particular interest from cases exposed to BCR::ABL1 tyrosine kinase inhibitors (TKI) during pregnancy

Case *n*°	Country and year of first report in Vigibase	Age	Cancer	Anticancer treatment and dose per day (if known)	Date relative start (months)	Trimester of exposure	Length of exposure (days)	Maternal ADR	Pregnancy events	Fetal‐newborn outcomes
i	USA 2018	UNK	CML	**Imatinib**	UNK	UNK	UNK	–	–	**Exomphalos**
ii	USA 2010	UNK	CML	**Imatinib 400 mg OD**	UNK	T2‐T3	~100 d	–	**Polyhydramnios**, premature rupture of membranes, preterm birth	–
iii	USA 2008	UNK	CML	**Imatinib**	UNK	UNK	UNK	–	Foetal growth restriction **hydrops fetalis**	Atrial septal defect, **death**
iv	Germany 2010	26	CML	**Imatinib 400 mg OD**	UNK	T1	UNK	–	Preterm birth (w26 to w32)	**Neonatal death, ascites, skin oedema, pericardial effusion**, hydrocephaly, cerebellar hypoplasia, spastic posture, premature baby, large atrial septum defect, transposition of aorta, neonatal respiratory failure
v	United Kingdom 2009	UNK	CML	**Imatinib 400 mg OD**	UNK	T1	UNK	–	–	Live birth, **exomphalos**, pulmonary hypoplasia, single functional kidney & kidney duplex, hemivertebra, shoulder deformation
vi	United Kingdom 2019	23	CML	**Imatinib 400 mg OD**	M6	T2‐T3	70‐80 d	Hepatic failure	**Polyhydramnios**	–
vii	South Africa 2009	UNK	CML	**Imatinib**	UNK	UNK	“Short term”	–	Ascites, induced abortion	**Death**
viii	Japan 2013	24	UNK	**Imatinib**	UNK	UNK	UNK	–	**Threatened preterm labour**	Resolution of threatened premature labour after 7 days
ix	USA 2010	UNK	UNK	**Imatinib 400 mg OD**	UNK	UNK	UNK	Oedema	**Threatened preterm labour**	–
x	Ireland 2003	UNK	CML	**Imatinib 400 mg OD** **interferon**	UNK	UNK	21 d UNK	–	Premature rupture of membranes	**Exomphalos**, renal agenesis, skeletal malformation/hemivertebra
xi	France 2016	25	AML	**Nilotinib 600 mg/d** **peginterferon alfa‐2a** **imatinib**	M1d1 M1d15 M6d1	T1 T1‐T2 T3	15 d 135 d 50d	–	**Polyhydramnios**, preterm birth (w34)	Duodenal stenosis
xii	Germany 2017	UNK	CML	**Nilotinib 300 mg OD**	M0d7	T1	UNK	**Pericardial effusion**	–	Disturbance in attention
xiii	Japan 2019	37	CML	**Nilotinib 400 mg/d** interferon gamma‐1a	M1d1 UNK	T1 UNK	35 d UNK	–	**Hydrops fetalis**, C‐section, preterm birth (w32.5)	Pulmonary hypoplasia, atelectasis neonatal, Atrioventricular septal defect, Lymphangioma, pleural effusion, newborn **death**
xiv	France 2021	36	CML	**Dasatinib 100 mg OD**	M6d6	T3	42 d	–	**Polyhydramnios**	Hydrothorax/pleural effusion
xv	United Kingdom 2020	23	CML	**Dasatinib 50 mg OD** **imatinib 500 mg OD**	T3 M6d17	T3	UNK 96 d	Hepatic failure	**Polyhydramnios**	Jaundice
xvi	Japan 2019	24	CML	**Dasatinib**	UNK	T1	UNK	Mirror syndrome	**Hydrops fetalis**, foetal movements decreased, C‐section, preterm birth (w33)	Thoracentesis
xvii	Korea 2016	UNK	UNK	**Dasatinib 100 mg OD**	UNK	T2‐T3	51 d	–	**Hydrops fetalis** IUGR preterm birth	Neonatal **death**, pleural effusion, pulmonary disorders
xviii	Oman 2011	27	UNK	**Dasatinib**	UNK	UNK	UNK	–	**Polyhydramnios**	–
xix	Australia 2015	UNK	UNK	**Dasatinib 100 mg OD**	UNK	UNK	UNK	–	**Hydrops fetalis**, preterm birth	Hepatomegaly
xx	Sweden 2019	UNK	CML	**Ponatinib 30 mg**	UNK	T1	~60 d	–	–	Hirschsprung's disease
xxi	Japan 2016	29	ALL	**Imatinib, cyclophosphamide, doxorubicin, vincristine, dexamethasone**	UNK	UNK	UNK	**Pleural effusion** pulmonary infarction	**Threatened preterm labour**	–
xxii	USA 2023	26	CML	**Asciminib 20 mg BID**	UNK	UNK	UNK	–	–	Normal newborn

ADR, adverse drug reaction; BID, bis in die; OD, Once Daily; USA, United States of America; UNK, Unknown; CML, Chronic Myeloid Leukemia; ALL, Acute Lymphoblastic Leukemia; AML, Acute Myeloid Leukemia; d, days; w, weeks; T, trimester.

Reports with maternofetal events of particular interest were summarized. Suspect anticancer drugs were in bold. ADRs of interest are in bold.

Three cases of exomphalos were also found in our cohort in association with imatinib (cases i, v, x), and none were observed with other BCR::ABL1 TKIs or anticancers. Two of these cases (cases v and x) were associated with hemivertebrae and renal abnormalities. One case of Hirschsprung's disease was associated with ponatinib use at T1 (case xx). One case of exposure to asciminib with an unspecified timing of exposure resulted in a normal pregnancy (case xxii).

## DISCUSSION

In this study, we analyzed, to our knowledge, the largest cohort of cases of maternofetal exposure to TKI targeting *BCR::ABL1* during pregnancy. We found that several TKI‐specific adverse outcomes, mainly hydrops fetalis, polyhydramnios, and threatened preterm labor, were more frequently reported for TKI treatments than for other anticancer drugs.

Among the 969 reports in the TKI‐exposed group, 580 (59.9%) had exposure without any pregnancy or fetal/newborn complication reported. These results are consistent with those previously reported by Pye *et al*.,^6^ with 50% of normal infants born after imatinib exposure during pregnancy among 125 pregnancies with known outcomes. In total, 9.6% resulted in infants with fetal abnormalities, including skeletal malformations, renal, respiratory, and gastrointestinal abnormalities. In the general population, major birth defect risk is about 2–3%.^19^ In our cohort of declared exposed pregnancies to TKI, we observed a proportion of 5.2% congenital malformations and 3.8% neonatal complications; fetal abnormalities occurred at similar rates than in literature about pregnancy on a TKI,^11^, ^20^ although direct comparisons between pharmacovigilance–derived proportions and incidence rates from systematically collected cohorts should be interpreted with caution. The only two cases of hemivertebrae reported in Vigibase were with imatinib, which were primarily described by Pye et *al*.^6^ We also found a higher rate of abortion, both spontaneous and induced, in the TKI‐exposed group compared to the other anticancer group. Spontaneous abortion rates are largely age‐related. In our study, the mean maternal age was not significantly different for BCR::ABL1 TKI compared to other anticancer drug exposure groups (28.5 years, SD = 12.6, vs 28.3 years, SD = 14.0, *P* = 0.42). However, with 8.2% of spontaneous abortion, our results are consistent with previous studies, reporting an equivalent incidence of spontaneous abortion to that in the non‐cancer population.^7^ In addition, since the risk of teratogenicity is well known to date, patients and physicians have adapted their behavior since the first publications. Discontinuation of TKIs before or very early after the onset of pregnancy, and limitation of the abortion strategy thanks to the use of early ultrasonography, are necessarily reflected in our results.

As a pioneer in the therapeutic arsenal, imatinib exposure during pregnancy has been more evaluated than second generation TKI in pregnancy for which there is less clinical experience. In the imatinib‐exposed population, threatened preterm labor was overreported and none of the cases had concomitant fetal/neonatal effusion, although the clinical relevance of this finding is uncertain. The interpretation of this outcome can be challenging due to the preterm birth rate in the TKI‐exposed group being close to that observed in the general population. There was also an increased rate of abortion, although this signal did not remain in the sensitivity analysis and could be linked to the specificities of the CML population with long treatment duration and increased proportion of patients exposed in the first trimester. Also, 3 cases of exomphalos have been reported in pregnancies with exposure to imatinib, and none with other anticancer drugs or other BCR::ABL1 TKI, although this analysis was not pre‐specified and imatinib was not associated with increased digestive malformations altogether.

Since the first TKI, imatinib, three new generations of TKIs are currently available in clinical practice: dasatinib, nilotinib, ponatinib, bosutinib, and more recently asciminib or radotinib approved in Korea. They all have their own safety and teratogenicity profile. Dasatinib is a dual BCR::ABL1/Src kinase inhibitor, crossing the placenta and leading to considerable levels in fetal plasma. It can provoke impairment of several key genes regulating angiogenesis, proliferation, survival, and apoptosis in a preclinical study.^21^ In the first trimester, dasatinib exposure has been associated with fetal hydrops and severe fetal bicytopenia^22^ or complex cardiopathy in a twin infant,^23^ but normal pregnancies have also been reported.^10^, ^20^ The largest cohort previously published with dasatinib concerned 46 women with known outcomes in the BMS Database,^7^ including seven infants with congenital abnormalities: one with renal tract issues (bilateral pyelocaliceal dilatation and kidney enlargement), two with hydrops fetalis, one with central nervous system abnormalities (encephalocele and craniosynostosis) and three others with fetal abnormalities unknown. In the current study, we reported the outcomes of 128 women exposed to dasatinib with an overreporting of hydrops fetalis and polyhydramnios, which is concordant with previous case series. In the whole cohort, we found 5 cases of fetal hydrops, of whom 3 were exposed to dasatinib (2.3%) including one categorized as a mirror syndrome. A case of hydrops fetalis was also found with nilotinib and imatinib, which were associated with concurrent cardiac malformation. Outside of pregnancy, dasatinib is known to induce pleural effusion by increasing endothelial permeability.^24^ These results can justify avoiding its use during pregnancy.

Few cases are published with nilotinib exposure during pregnancy,^10^, ^11^, ^20^ with serum and placenta analysis mentioning reduced placental crossing beyond week 16 and no major adverse events.^12^ In our cohort of 220 patients with nilotinib exposure during pregnancy, no specific overreported AE has been found.

Regarding ponatinib, data is also lacking.^12^ Among 11 reports associated with ponatinib exposure, we found one case of Hirschsprung's disease with exposure during the first trimester.

This study has some other limitations mainly linked to the disproportionality pharmacovigilance approach with inconsistencies in reporting and collection of information.^25^, ^26^ The trimester and duration of exposure to TKIs during pregnancy are major issues in the occurrence of fetal and obstetric complications, which are unfortunately not accurately assessed for each case reported in the database.

This could limit the conclusions being drawn about the incidence of these events in the general population. However, apart from abortions, sensitivity analyses carried out among more homogenous subgroups consistently reaffirmed our primary findings.

## CONCLUSIONS

The findings of our disproportionality analysis study present substantial evidence that BCR::ABL1 TKI exposure is associated with the occurrence of pregnancy complications such as hydrops fetalis, polyhydramnios among fetuses or newborns, which were more specific to dasatinib. Few exomphalos complications have been reported with imatinib, without increased digestive malformation overall. Imatinib and nilotinib appear to be associated with fewer maternal‐fetal complications. However, further studies are needed to assess whether their use during pregnancy could be considered with interferon‐alpha, in cases where delay in maternal treatment could lead to serious outcomes, particularly in cases of highly proliferative disease, accelerated phase, or blastic crisis.

## FUNDING

PG was funded by the academic program “Contrats ED: Programme blanc Institut Curie PSL” during this study. The Institut Curie RT2L research group (PG, ASH, FJ, ED, RK, FC, EL, FR) was supported by the academic program “SHS INCa”, and is part of a research project on young women funded by Monoprix*.

## CONFLICT OF INTEREST

Aurélie Cabannes‐Hamy reports advisory board for Novartis, Amgen, and Gilead‐Kite. Kevin Bihan declares reporting consulting fees for Medinspire. Sylvain Choquet declares fees from Abbvie, Amgen, Astra Zeneca, Atara, Gilead‐Kite, Janssen, Lilly, Novartis, Pierre Fabre, and Takeda. Paul Gougis reports consulting fees for BMS, an academic grant from Sanofi, and travel accommodation by Eisai. All other authors declared no competing interests for this work.

## AUTHOR CONTRIBUTIONS

ACH, RK, PG, FC, EL, and ASH wrote the manuscript; PG, KB, ED, FR, and ASH designed the research; RK, PG, FJ, PR, SC, MU, and LS performed the research; RK, ED, and PG analyzed the data.

## Supporting information


Appendix S1.

## Data Availability

Publicly available datasets were analyzed in this study. The data can be obtained from: www.vigiaccess.org. This study has not been published or presented elsewhere. The information does not represent the opinion of the Uppsala Medical Center or the World Health Organization.
