# Supplementary material for: BCR::ABL1 Tyrosine Kinase Inhibitors During Pregnancy, a Disproportionality Analysis of Vigibase
Source: Clin Pharmacol Ther. 2025 May 29;118(3):705–14. doi: 10.1002/cpt.3730 (PMC12355020; doi:10.1002/cpt.3730)
Supplement: Supplementary file 1 — Appendix S1. [file CPT-118-705-s001.docx]

Supplemental Materials

[Supplementary Figures 3](#_Toc192167972)

[Figure S1. Directed Acyclic Graph for assessment of confounding factors. 3](#_Toc192167973)

[Figure S2. Characteristics of reports in study population. 4](#_Toc192167974)

[Figure S3. UpSet plot of the reporting of anticancer drugs in the BCR-ABL Tyrosine Kinase Inhibitors exposed group (n=969). 5](#_Toc192167975)

[Figure S4. Sensitivity analysis within the subpopulation treated with single-class drugs. 6](#_Toc192167976)

[Figure S5. UpSet plot of the co-occurrence of the main maternofetal adverse events of interest with BCR-ABL Tyrosine Kinase Inhibitors exposure. 7](#_Toc192167977)

[Figure S6. Multivariable analysis. 8](#_Toc192167978)

[Figure S7. Sensitivity analysis within the subpopulation of patients receiving only anticancer in single class (n=1697). 9](#_Toc192167979)

[Figure S8. Sensitivity analysis within the subpopulation of patients with chronic myeloid leukemia. 10](#_Toc192167980)

[Figure S9. Sensitivity analysis within patients with no chronic myeloid leukemia. 11](#_Toc192167981)

[Supplementary Tables 12](#_Toc192167982)

[Table S1. Detail of VigiBase query. 12](#_Toc192167983)

[Table S2. Terms corrected. 13](#_Toc192167984)

[Table S3. MedDRA Preferred Terms used to qualify reports’ exposure type. 14](#_Toc192167985)

[Table S4. 45 individual materno-fetal adverse outcomes explored 15](#_Toc192167986)

[Table S5. Terms deemed not clinically significant. 16](#_Toc192167987)

[Table S6. The Reporting of a Disproportionality Analysis for Drug Safety Signal Detection Using Individual Case Safety Reports in PharmacoVigilance (READUS‑PV) checklist 17](#_Toc192167988)

[Table S7. The Strengthening the Reporting of Observational Studies in Epidemiology (STROBE) checklist: guidelines for reporting observational studies. 22](#_Toc192167989)

[Table S8. Pregnancy or fetal/newborn adverse outcomes in the TKI group and in the other anticancer group. 26](#_Toc192167990)

[Table S9. Subgroups analysis by types of TKI molecules 28](#_Toc192167991)

[Supplemental Methods 32](#_Toc192167992)

[Identification of reports addressing mother vs fetal/newborn 32](#_Toc192167993)

[Position of the problem 32](#_Toc192167994)

[Scoring system for each report 32](#_Toc192167995)

[Final attribution 33](#_Toc192167996)

[Deduplication algorithm 34](#_Toc192167997)

[Problem position 34](#_Toc192167998)

[Initial selection 34](#_Toc192167999)

[Fusion of duplicates 36](#_Toc192168000)

[Fusion of dyads 37](#_Toc192168001)

[Position of the problem 37](#_Toc192168002)

[Inclusion criteria 37](#_Toc192168003)

[Exclusion criteria 37](#_Toc192168004)

# Supplementary Figures

## Figure S1. Directed Acyclic Graph for assessment of confounding factors.

Directed Acyclic Graph for the mitigation of main confounding factors of reporting (constructed using DAGitty https://www.dagitty.net). “▷” is the exposure and “I” is the outcome.


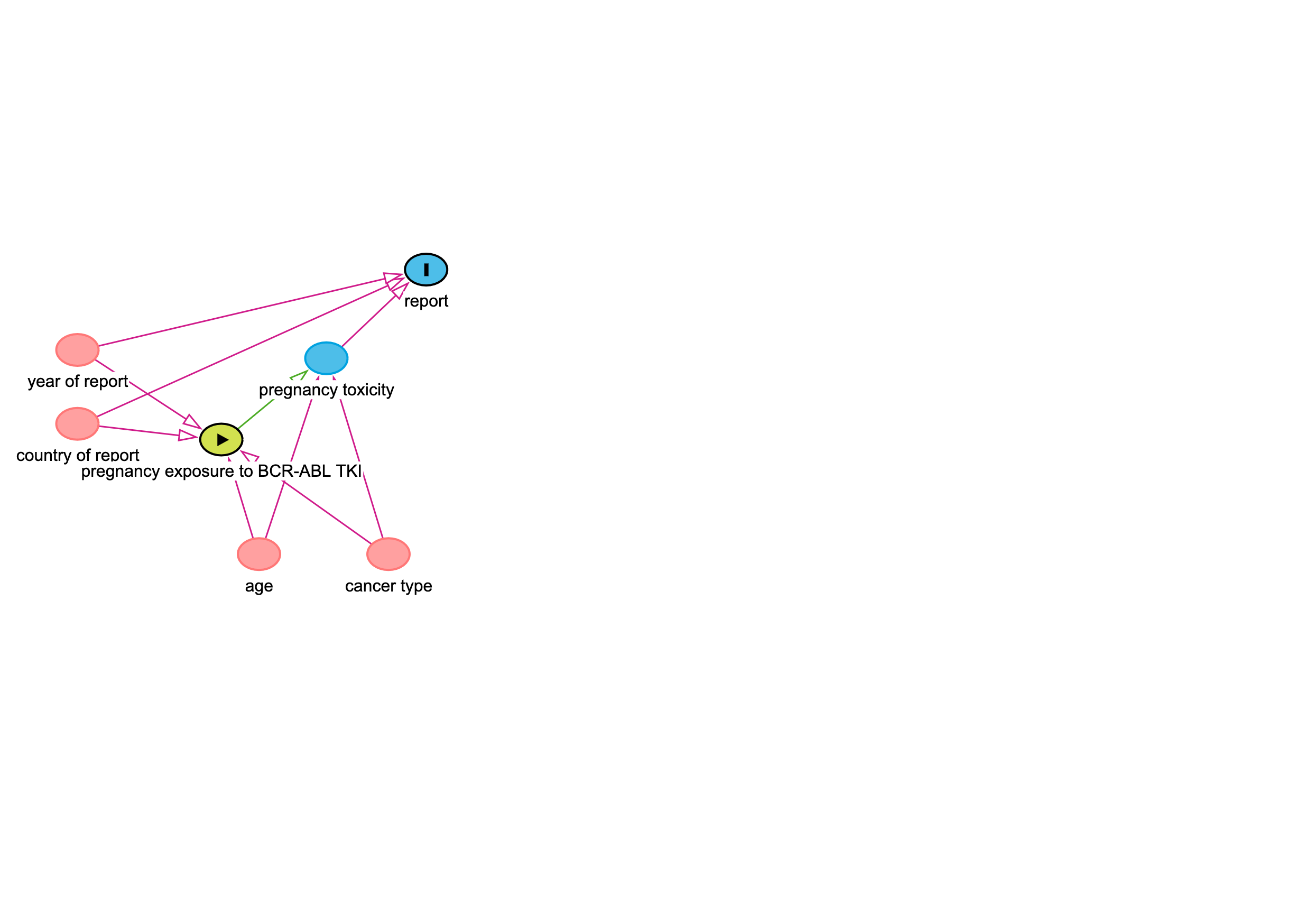


## Figure S2. Characteristics of reports in study population.

Characteristics of reports in study population for BCR-ABL Tyrosine Kinase Inhibitors exposure group compared to exposure to other anticancers. Panel A represent the age at diagnosis, panel B the year of report and panel C the cancer type identified within report. Two or more cancers could be diagnosed in a single report.


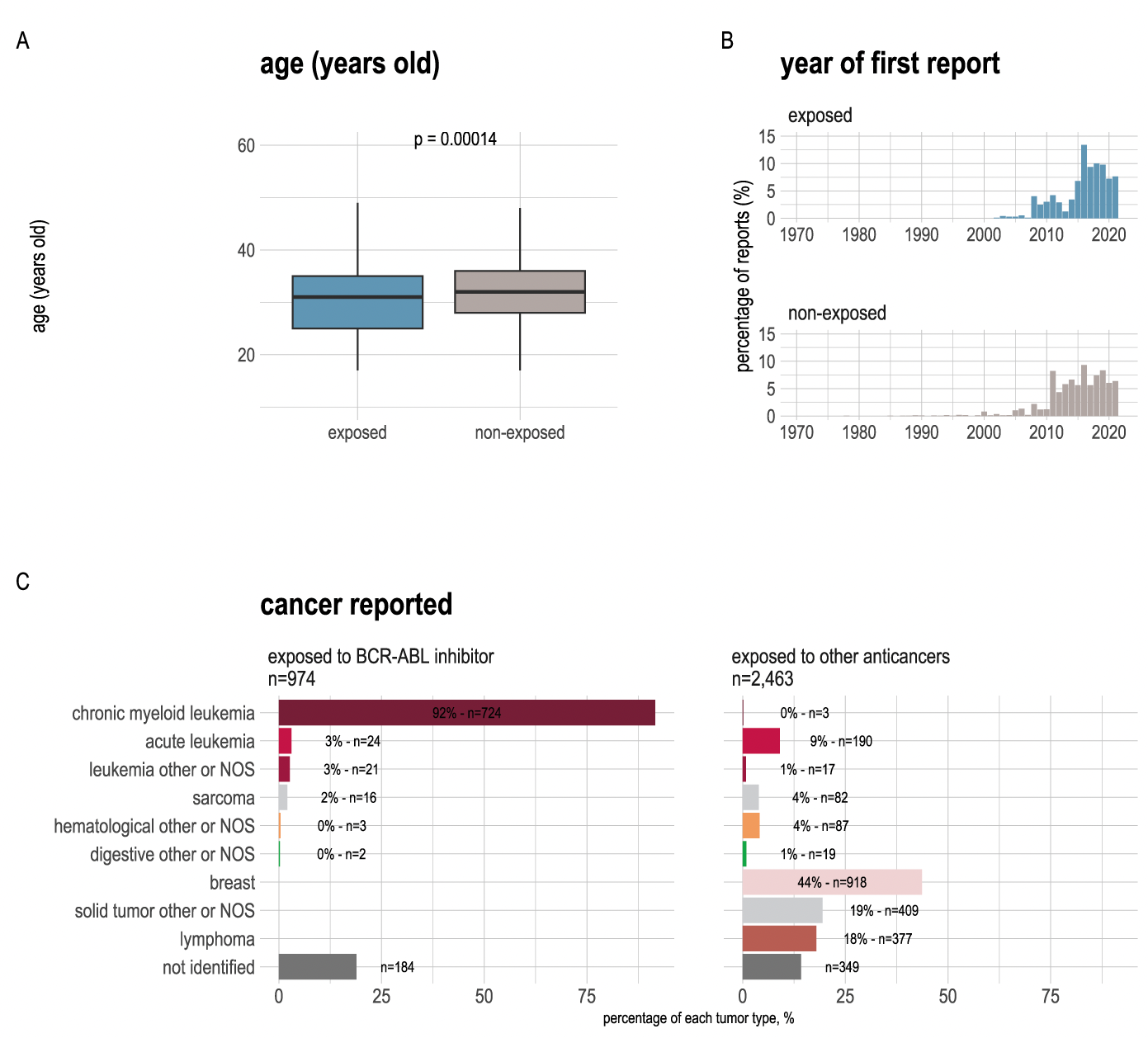


## Figure S3. UpSet plot of the reporting of anticancer drugs in the BCR-ABL Tyrosine Kinase Inhibitors exposed group (n=969).

The intersection size represents the number of reports for which the modality of intersection below is found. The Set size is the number of reports for which the treatment has been reported. Intersection size for reports with BCR-ABL TKI-only are in purple (total n=896), and reports with combination with other agents (cytotoxic chemotherapies, or Interferon alpha) are displayed as orange (n=40) and gray bars (n=33) respectively.


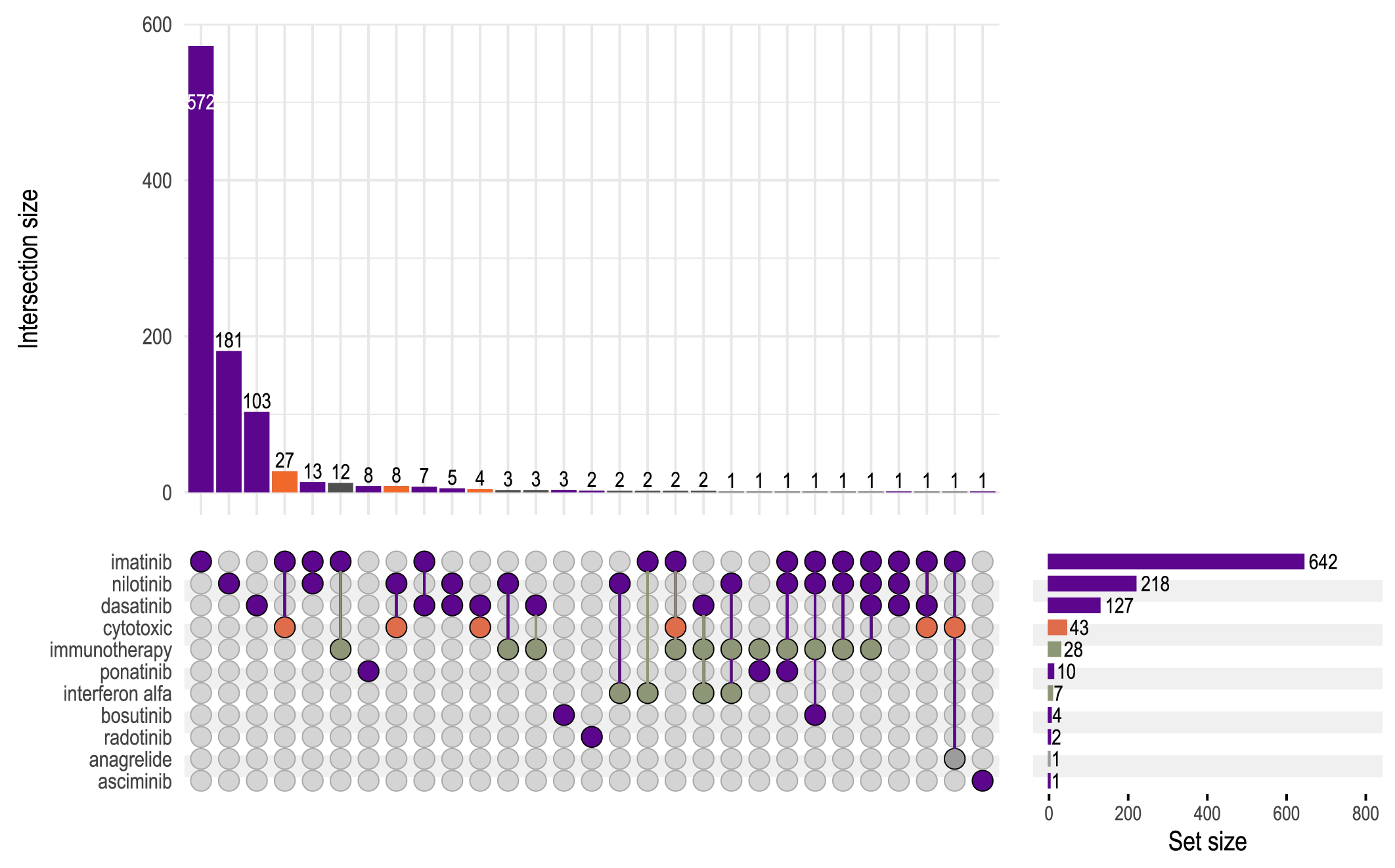


## Figure S4. Sensitivity analysis within the subpopulation treated with single-class drugs.

Description of the number of pregnancy and/or fetal or newborn outcome among patients receiving BCR-ABL Tyrosine Kinase Inhibitors monotherapy (N=896) or in combination with other anticancers (N=73).

Bars represent the percentage of each outcome divided by the total number of reports. Counts are annotated. Among 45 pre-specified maternal-fetal adverse outcomes types, we represented toxicities for which at least one case was found.

Abbreviations: HT: Hypertension; NOS: Not Otherwise Specified; TKI: Tyrosine Kinase Inhibitor

BCR-ABL TKI

BCR-ABL TKI

BCR-ABL TKI

## Figure S5. UpSet plot of the co-occurrence of the main maternofetal adverse events of interest with BCR-ABL Tyrosine Kinase Inhibitors exposure.

The intersection size represents the number of reports for which the modality of intersection below is found. The Set size is the number of reports for which the treatment has been reported.


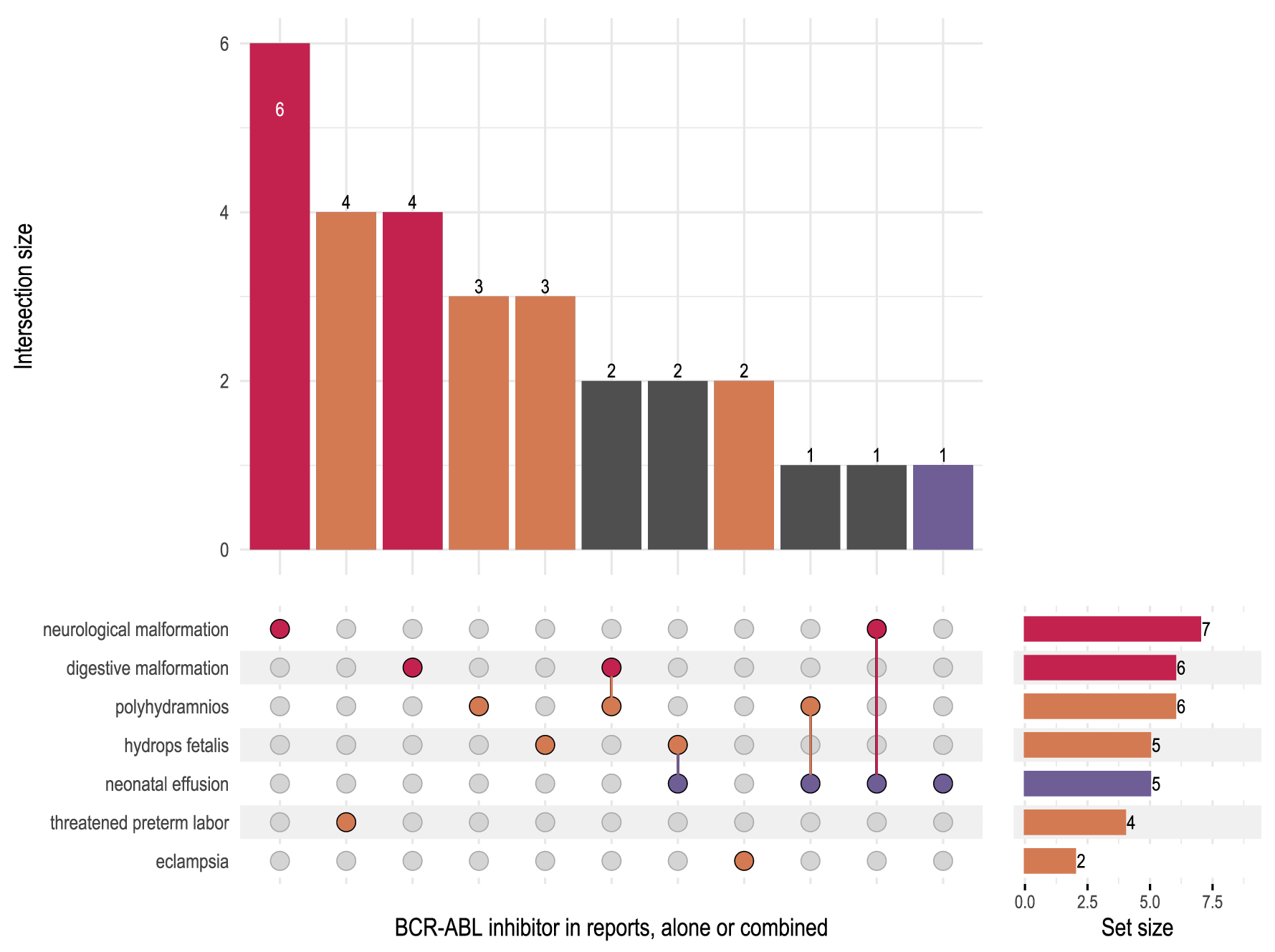


## Figure S6. Multivariable analysis.

BCR-ABL TKI

Risk of adverse pregnancy and fetal/newborn outcomes with exposure to BCR-ABL Tyrosine Kinase Inhibitors (TKI) compared to other anticancers.

The adjustment was made on the year and country of the report, patient’s age and cancer type. Analysis was limited to adverse outcomes with 2 or more occurrences in the TKI-exposed group. ORs are shown with [CI95%].

P-values: *p-value≤0.05, **p-value≤0.01, ***p-value≤0.001.

Abbreviations: aOR: adjusted odds ratio; CI: Confidence Interval; HT: hypertension; NOS: not otherwise specified; Ntox: number exposed to BCR-ABL with toxicity; TKI: Tyrosine Kinase Inhibitor


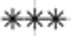


**aOR [CI95%]**

**Ntox**

**maternal-fetal adverse outcomes types**


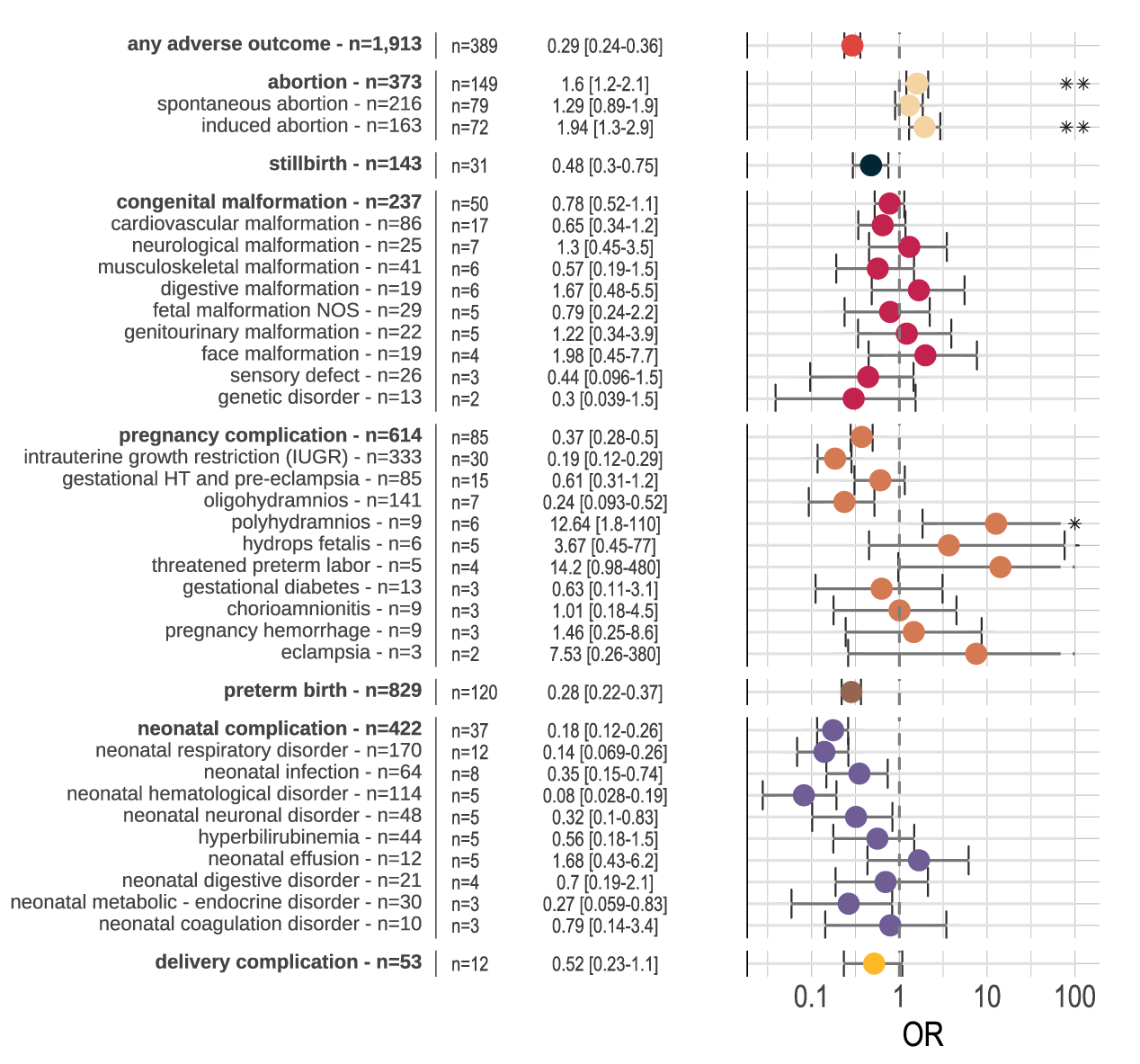


## Figure S7. Sensitivity analysis within the subpopulation of patients receiving only anticancer in single class (n=1697).

Description and reporting odds ratio (ROR) of pregnancy and fetal/newborn adverse outcomes with exposure to BCR-ABL tyrosine kinase inhibitors (TKI) compared to exposure to other anticancer drugs as single class.

Bars represent the percentage of each outcome divided by the total number of reports. Counts are annotated. RORs are displayed as logarithmic data for the purpose of data visualization. Among 45 pre-specified maternal-fetal adverse outcomes types, we represented toxicities for which at least one case was found in the TKI-exposed group.

P-values: *p-value≤0.05, **p-value≤0.01, ***p-value≤0.001.


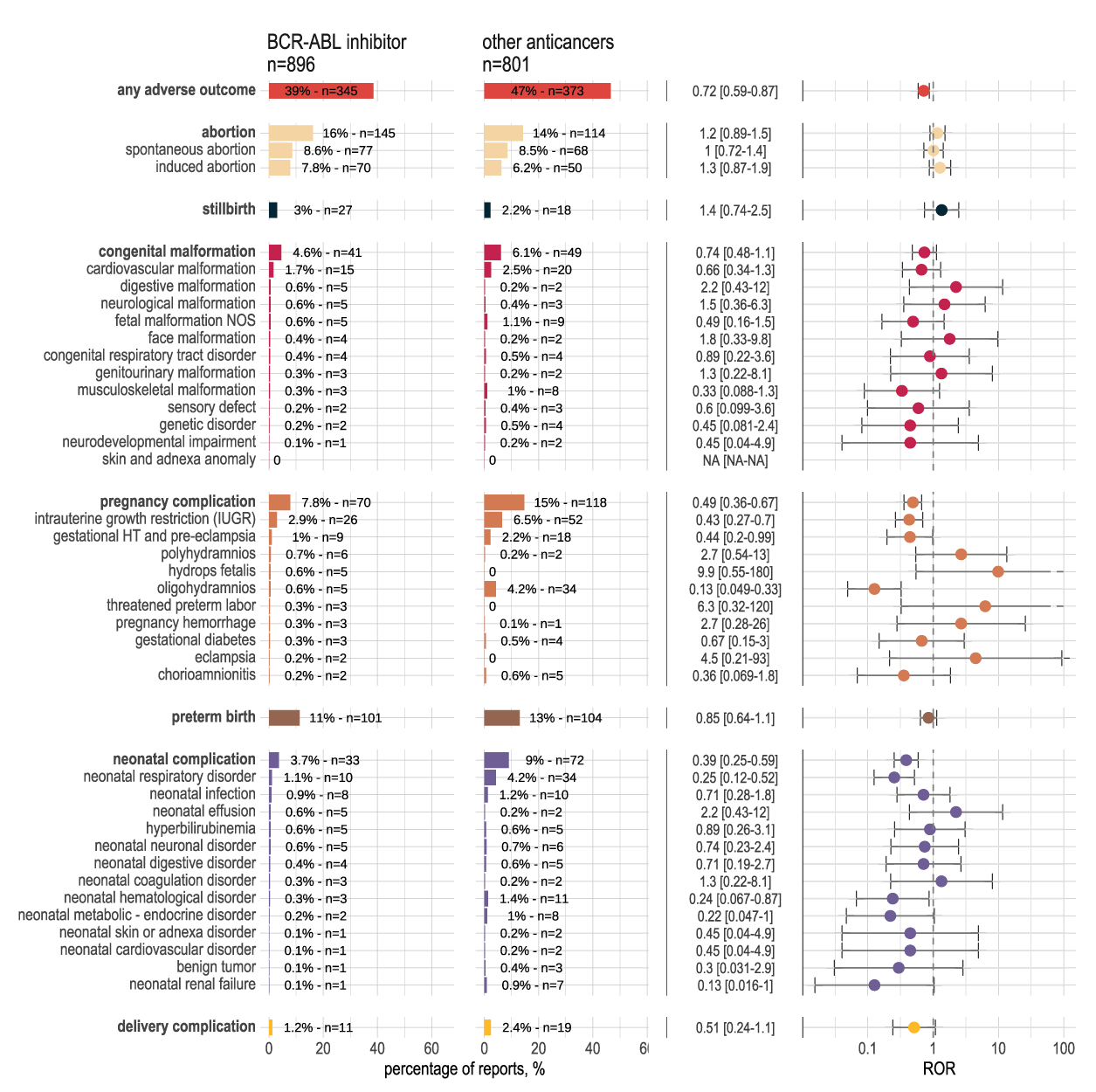
Abbreviations: NOS: Not Otherwise Specified; HT: Hypertension; ROR: Reported Odds Ratio; TKI: Tyrosine Kinase Inhibitor

BCR-ABL TKI

## Figure S8. Sensitivity analysis within the subpopulation of patients with chronic myeloid leukemia.

Description and reporting odds ratio (ROR) of pregnancy and fetal/newborn adverse outcomes with exposure to BCR-ABL tyrosine kinase inhibitors (TKI) individually in the population with an identified chronic myeloid leukemia.

Bars represent the percentage of each outcome divided by the total number of reports. Counts are annotated. RORs are displayed as logarithmic data for the purpose of data visualization. P-values: *p-value≤0.05, **p-value≤0.01, ***p-value≤0.001. When the N observed was one, the result was not considered significant.

Abbreviations: HT: Hypertension; NOS: Not Otherwise Specified; ROR: Reported Odds Ratio


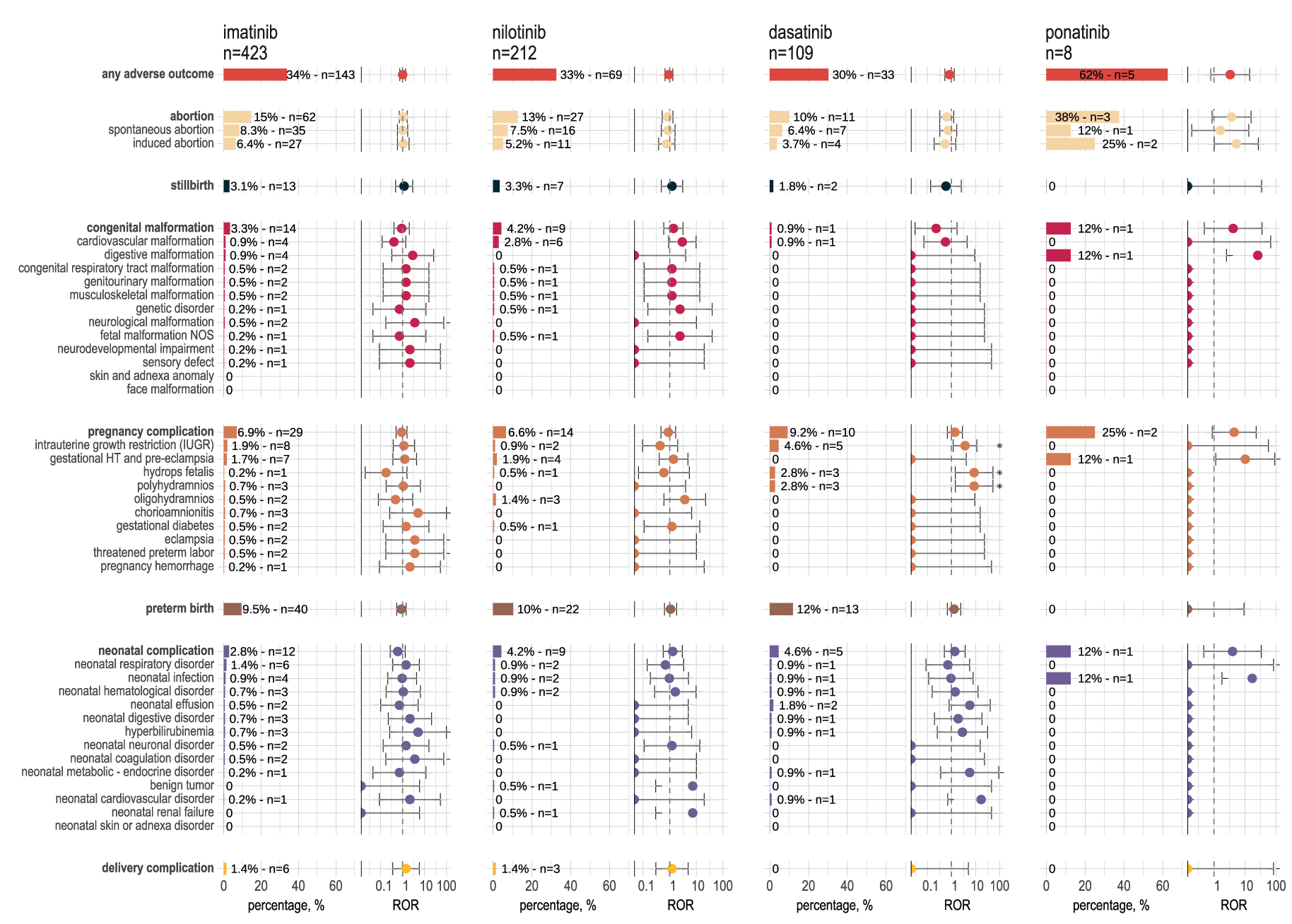


## Figure S9. Sensitivity analysis within patients with no chronic myeloid leukemia.

Description and reporting odds ratio (ROR) of pregnancy and fetal/newborn adverse outcomes with exposure to BCR-ABL tyrosine kinase inhibitors (TKI) compared to exposure to other anticancer drugs in the population with cancers other than chronic myeloid leukemia.

Bars represent the percentage of each outcome divided by the total number of reports. Counts are annotated. RORs are displayed as logarithmic data for the purpose of data visualization. Among 45 pre-specified maternal-fetal adverse outcomes types, we represented toxicities for which at least one case was found in the TKI-exposed group. P-values: *p-value≤0.05, **p-value≤0.01, ***p-value≤0.001. When the N observed was one, the result was not considered significant.

Abbreviations: NOS: Not Otherwise Specified; HT: Hypertension; ROR: Reported Odds Ratio; TKI: Tyrosine Kinase Inhibitor; ROR


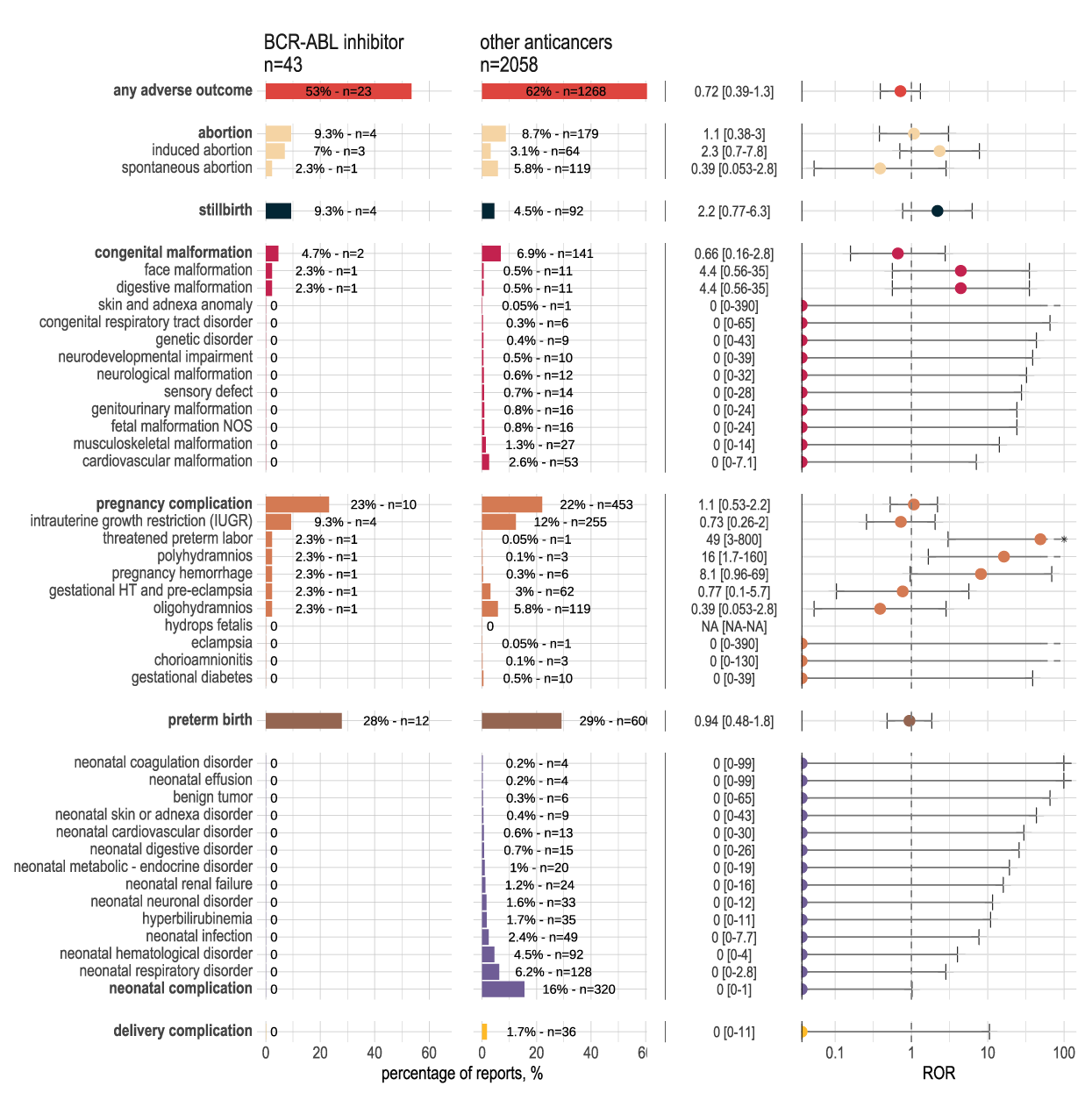


BCR-ABL TKI

# Supplementary Tables

## Table S1. Detail of VigiBase query.

MedDRA terms used for the initial identification of reports.

| VigiBase data set date | 01/01/2024 |  |
| --- | --- | --- |
| MedDRA version | MedDRA 26.1 (English) |  |
| Search criteria for VigiBase extraction | drugs (ATC group) | L01 ANTINEOPLASTIC AGENTS |
|  | reaction and MedDRA terms | Pregnancy, puerperium and perinatal conditions (SOC) |
|  |  | Fetal and neonatal investigations (HLGT) |
|  |  | Neonatal and perinatal conditions (HLGT) |
|  |  | Neonatal respiratory disorders (HLGT) |
|  |  | Exposures associated with pregnancy, delivery and lactation (HLT) |
|  |  | Fetal therapeutic procedures (HLT) |
|  |  | Induced abortions (HLT) |
|  |  | Obstetric therapeutic procedures (HLT) |
| Number of reports from VigiBase extraction | 10,832 deduplicated cases match your search |  |

## Table S2. Terms corrected.

Terms corrected from initial identification of reports

| MedDRA preferred terms not always associated with pregnancy | |
| --- | --- |
| MedDRA preferred term | mapped as |
| Pelvic girdle pain | Pregnancy symptom |
| Morning sickness | Pregnancy symptom |
| Ghost pregnancy (pseudo embarazo) | Pregnancy symptom |
| Bronchopulmonary dysplasia | Neonatal hypoxic conditions |
| Brief resolved unexplained event | Neonatal hypoxic conditions |
|  |  |
| MedDRA mapping problem |  |
| reported term | mapped as |
| Utero, contracciones | Uterine contractions during pregnancy* |
|  |  |
| Term wrongly encoded |  |
| reported term | mapped as |
| 10049058 | HELLP syndrome** |
| Cervical dilatation | translation problem, cervical dilatation for a neck edema |
|  |  |
| Terms wrongly associated with anticancer drugs | |
| term | mapped as |
| MK-8962 MK-8328 Mk-8415 Mk-9384 | Pembrolizumab |
| Terms too unspecific for classification | |
| term | mapped as |
| Monoclonal antibodies  Anticuerpo monoclonal (sin especificar) | antineoplastic monoclonal antibodies |
| Chemotherapeutics  Chemotherapy drugs | antineoplastic agents |
| Chemotherapy nos | antineoplastic and immunomodulating agents |

| *cases of uterus contractions without pregnancy |
| --- |
| **Italian reports link this term to HELLP syndrome, although none are linked to a pregnancy |

## Table S3. MedDRA Preferred Terms used to qualify reports’ exposure type.

MedDRA preferred terms used in reports for the identification of the timing and modality of exposure.

| Exposure type | Preferred Terms |
| --- | --- |
| exposure during pregnancy | Exposure during pregnancy |
|  | First trimester pregnancy |
|  | Foetal exposure during delivery |
|  | Foetal exposure during pregnancy |
|  | High risk pregnancy |
|  | Maternal exposure during delivery |
|  | Maternal exposure during pregnancy |
|  | Pregnancy |
|  | Pregnancy on contraceptive |
|  | Pregnancy on oral contraceptive |
|  | Pregnancy with advanced maternal age |
|  | Pregnancy with contraceptive device |
|  | Pregnancy with injectable contraceptive |
|  | Unintended pregnancy |
|  | Unwanted pregnancy |
| exposure before pregnancy | Drug exposure before pregnancy |
|  | Maternal exposure before pregnancy |
| exposure via breast milk | Exposure via breast milk |
|  | Maternal exposure during breast feeding |
| exposure via semen | Exposure via body fluid |
|  | Exposure via father |
|  | Exposure via partner |
|  | Maternal exposure via partner during pregnancy |
|  | Paternal drugs affecting foetus |
|  | Paternal exposure before pregnancy |
|  | Paternal exposure during pregnancy |
|  | Paternal exposure timing unspecified |
|  | Pregnancy of partner |
| exposure via skin | Accidental exposure to product |
|  | Exposure via direct contact |
|  | Exposure via skin contact |
|  | Occupational exposure to product |

## Table S4. 45 individual materno-fetal adverse outcomes explored

| **outcome category** | **outcome type** |
| --- | --- |
| **abortion** | spontaneous abortion |
|  | induced abortion |
| **stillbirth** | fetal death - stillbirth |
| **congenital malformation** | cardiovascular malformation |
|  | musculoskeletal malformation |
|  | neurological malformation |
|  | fetal malformation not otherwise specified (NOS) |
|  | sensory defect |
|  | digestive malformation |
|  | face malformation |
|  | genitourinary malformation |
|  | genetic disorder |
|  | congenital respiratory tract malformation |
|  | multiple malformation or malformation syndrome |
|  | skin and adnexa anomaly |
| **pregnancy complication** | intrauterine growth restriction (IUGR) |
|  | oligohydramnios |
|  | gestational hypertension and pre-eclampsia |
|  | pregnancy complication NOS |
|  | placental disorder other |
|  | fetal disorder NOS |
|  | chorioamnionitis |
|  | gestational diabetes |
|  | polyhydramnios |
|  | pregnancy hemorrhage |
|  | hydrops fetalis |
|  | threatened preterm labor |
|  | eclampsia |
|  | HELLP syndrome |
| **preterm birth** | preterm birth |
| **neonatal complication** | neonatal respiratory disorder |
|  | neonatal hematological disorder |
|  | neonatal infection |
|  | hyperbilirubinemia |
|  | neonatal neuronal disorder |
|  | neonatal disorder other or NOS |
|  | neonatal metabolic - endocrine disorder |
|  | neonatal immune disorder |
|  | neonatal digestive disorder |
|  | neonatal cardiovascular disorder |
|  | neonatal coagulation disorder |
|  | benign tumor |
|  | malignant tumor |
|  | neonatal sensory disorder |
| **delivery complication** | delivery complication |

## Table S5. Terms deemed not clinically significant.

MedDRA preferred terms deemed not clinically significant when reported alone

| **preferred term reported deemed not clinically significant when reported alone** | **number of occurrences in the whole cohort**  **(n= 3,479)** |
| --- | --- |
| Foetal heart rate abnormal | 12 |
| Foetal hypokinesia | 11 |
| Uterine contractions during pregnancy | 8 |
| Poor feeding infant | 5 |
| Weight decrease neonatal | 5 |
| Foetal heart rate disorder | 3 |
| Large for dates baby | 3 |
| Bradycardia neonatal | 2 |
| Foetal heart rate deceleration abnormality | 2 |
| Foetal heart rate increased | 2 |
| Postmature baby | 2 |
| Uterine contractions abnormal | 2 |
| Agitation neonatal | 1 |
| Foetal arrhythmia | 1 |
| Foetal heart rate decreased | 1 |
| Foetal monitoring abnormal | 1 |
| Phimosis | 1 |
| Poor weight gain neonatal | 1 |
| Tachycardia foetal | 1 |

## Table S6. The Reporting of a Disproportionality Analysis for Drug Safety Signal Detection Using Individual Case Safety Reports in PharmacoVigilance (READUS‑PV) checklist

|  | Item No | Recommendation | Presence in manuscrit | Explication |
| --- | --- | --- | --- | --- |
| Title | 1a | If disproportionality analyses are a prominent component of the published study, the study should be identified as a “disproportionality analysis.” The type of data and name of the database(s) should be specified. | yes | “BCR::ABL1 Tyrosine Kinase Inhibitors during pregnancy, a disproportionality analysis of Vigibase” |
|  | 1b | Report the name of adverse event(s) and/or drug(s) under study, when applicable. | yes | “BCR::ABL1 Tyrosine Kinase Inhibitors during pregnancy” |
| Introduction |  |  |  |  |
| Background | 2a | Describe the drug(s) and its utilization, the nature of the adverse event(s) under study and its frequency,  and the existing knowledge on the drug–event combination. | yes | “Since the early 2000s, the introduction of BCR::ABL1 tyrosine kinase inhibitors (TKIs) has dramatically improved the outcome of patients with CML”  “In a study of 125 pregnant women taking imatinib, 50% had normal deliveries, but 12 infants (9.6%) were born with congenital abnormalities” |
|  | 2b | Specify the rationale for performing the analysis, e.g., as part of routine pharmacovigilance, to investigate an overall safety profile, or to assess a prespecified hypothesis. | yes | “The World Health Organization VigiBase, a global pharmacovigilance database, contains over 36 million individual case safety reports of adverse drug reactions to date from more than 130 countries since 1967. This database is a valuable resource in uncovering new adverse drug reactions.^14^” |
|  | 2c | Explain why individual case safety report databases and disproportionality analysis are suitable to fill the  knowledge gap. | yes | “This database is a valuable resource in uncovering new adverse drug reactions.^14^ In onco-hematology, good pharmacovigilance practice guidelines^15^ stipulate medical professionals should closely observe and report any instances of pregnancy both during clinical trials or regular treatment.” |
| Objectives | 3 | State specific objectives, identifying the adverse event(s), the drug(s), and the reference group, including any pre-specified hypothesis, if applicable. | yes | “The main objective of this study was to perform a disproportionality analysis of pregnancy and fetal or newborn adverse outcomes after exposure to BCR::ABL1 TKI compared to exposure to other anticancer drugs. Secondary analysis included the investigation of molecule-specific toxicities.” |
| Methods |  |  |  |  |
| Study design | 4a | Identify the study (i.e., “disproportionality analysis”) and the type of data used (e.g., “individual case safety reports”). | yes | “This cohort study utilizes pharmacovigilance individual case safety reports”  “We performed a case/non-case disproportionality analysis to evaluate the association between maternal and fetal/newborn adverse outcomes and exposure to TKI compared to exposure to other anticancer agents” |
|  | 4b | Provide an outline of the entire study design, including primary and sensitivity analyses performed, and other designs such as case-by-case analysis or literature review. | yes | “We performed a case/non-case disproportionality analysis to evaluate the association between maternal and fetal/newborn adverse outcomes and exposure to TKI compared to exposure to other anticancer agents within reports of patients with cancer and anticancer drug exposure during pregnancy."  The **Statistical analysis** **and** **Mitigation of biases and confounding factors** sections provide further details on the primary and sensitivity analyses performed |
| Data description, access,  and pre-processing | 5a | Specify the name of the database(s), the database(s) custodian, and the coverage. Specify the type/number of drugs included within the database and the thesaurus,taxonomies, or ontologies used for coding drugs and events. | yes | "This cohort study utilizes pharmacovigilance individual case safety reports from VigiBase, the WHO global database of reported potential side effects of medicinal products, which is developed and maintained by the Uppsala Monitoring Centre."  "Anticancer drugs were any drugs from the 'antineoplastic' Anatomical Therapeutic Classification (ATC) group L01." |
|  | 5b | Specify the extraction dates and describe and justify all choices used for data pre-processing, including any  data transformation or exclusion, if appropriate. | yes | "VigiBase was queried on January 1st, 2024, with Medical Dictionary for Regulatory Activities (MedDRA) version 26.1." "We ensured that only reports mentioning pregnancy-associated conditions/exposure were retained by discarding reports with terms secondarily associated with pregnancy. Only reports with terms primarily associated with pregnancy as a main SOC, HLGT, or HLT were retained."  *“Reports were then analyzed to discard:*   - *Reports with no mention of a cancer diagnosis or with an antineoplastic drug from the L01 ATC group prescribed for a non-cancer indication (e.g., prescription of methotrexate for rheumatoid arthritis or of alemtuzumab for multiple sclerosis);* - *Reports with drug mapping problems or adverse event mapping problems (Table S2).* - *Dyads, i.e., maternal and fetal reports referring to the same case, were detected using an in-house information-entropy based algorithm and merged (cf. Supplemental Methods)."*   "Reports with notification of a term associated with exposure via skin or semen were excluded. Reports with exposure via breast milk or before pregnancy and no specific mention of exposure during pregnancy were also discarded." |
| Variables definition | 6a | Describe the study population, including any restriction. | yes | "We ensured that only reports mentioning pregnancy-associated conditions/exposure were retained by discarding reports with terms secondarily associated with pregnancy. Only reports with terms primarily associated with pregnancy as a main SOC, HLGT, or HLT were retained."  *"Reports were then analyzed to discard:*   - *Reports with no mention of a cancer diagnosis or with an antineoplastic drug from the L01 ATC group prescribed for a non-cancer indication (e.g., prescription of methotrexate for rheumatoid arthritis or of alemtuzumab for multiple sclerosis);* - *Reports with drug mapping problems or adverse event mapping problems (Table S2).* - *Dyads, i.e., maternal and fetal reports referring to the same case, were detected using an in-house information-entropy based algorithm and merged (cf. Supplemental Methods)."* |
|  | 6b | Describe the nature and the meaning of key variables assessed in the work. | yes | "Cases were reports with mention of a maternal and fetal/newborn adverse events categorized from MedDRA preferred terms in VigiBase. They constituted 45 individual maternal-fetal adverse outcomes regrouped into seven categories for the purposes of this study: Abortion (induced and spontaneous); Stillbirth/fetal death; Congenital malformation; Pregnancy complication; Preterm birth; Neonatal complication; and Delivery complication.”  "The reporting odds ratio (ROR) was defined as the ratio of the odds of the adverse pregnancy or fetal/newborn outcome of interest with exposure to TKI to the odds with exposure to other anticancer drugs." |
|  | 6c | Specify and justify any grouping of drugs or events. For drugs, specify and justify whether active ingredients/trade names/salts were considered and/or the selected role. |  | We considered the following drugs to be TKI targeting BCR::ABL1: imatinib, nilotinib, dasatinib, ponatinib, bosutinib, asciminib and radotinib. Any report from the study analysis with a mention of a BCR::ABL1 TKI drug was qualified as a 'TKI exposure group'."  "Reports with other anticancers and no mention of BCR::ABL1 TKI were qualified as the 'exposure to other anticancers' group."  *"Cases were reports with mention of a maternal and fetal/newborn adverse events categorized from MedDRA preferred terms in VigiBase. They constituted 45 individual maternal-fetal adverse outcomes regrouped into seven categories for the purposes of this study”* |
|  | 6d | Describe any additional data source used, the type of data, and how they interact with individual case safety reports. | not applicable |  |
| Statistical methods | 7a | Present any descriptive analysis performed, specifying variables investigated, statistical tests, and significance thresholds. | yes | "The study population is described in terms of frequencies for qualitative variables or medians and interquartile range [IQR] for quantitative variables." "Associations between categorical variables were assessed with Fisher tests. P-values of less than 0.05 were considered statistically significant." |
|  | 7b | Describe the measure(s) selected for the disproportionality analysis including any threshold used to identify  signals of disproportionate reporting. Explain the reason for this choice if applicable. | yes | "A signal was considered to be present when statistically significant disproportionality, with the lower end of the ROR confident interval, ROR025, was over 1 and the number of occurrences was 2 or more." |
|  | 7c | Clearly describe any sensitivity analysis and any tool to control confounding, including any restriction,  subgroup, stratification, adjustment, or interaction. | yes | "we conducted sensitivity/subgroup analyses on reports for which a single class of treatment was used (BCR::ABL1 TKI in the TKI-exposed group). For this analysis, any report with a combination of drug classes, such as cytotoxic+TKI, was discarded."  "To identify specific effects of each TKIs, we conducted a disproportionality analysis for each TKI molecule independently with substantial numbers (N>10): imatinib, nilotinib, dasatinib and ponatinib."  "Another sensitivity analysis was done within the population of reports for which a chronic myeloid leukemia was identified. To assess the impact of CML on our results, we also evaluated the impact of BCR::ABL1 TKIs excluding reports with a diagnosis of CML."  *"To limit the impact of biases, we also identified confounding variables using a Directed Acyclic Graph (Figure S1). The 'year of report', the 'country of report', 'patient’s age' and the 'cancer type' were main variables that needed adjustment to limit confounding factors."*  *"The odds ratio for the risk of each toxicity was then evaluated using a multivariate analysis by logistic regression with adjustment on these variables. Missing data were grouped within a single level of value for each variable."* |
|  | 7d | Specify the variables and methods used for the case-by-case analysis, including any algorithm or criteria  used to assess causality, if performed. | No | *No causality assessment* |
|  | 7e | Specify any statistical methods used for other data sources. | not applicable |  |
| Results |  |  |  |  |
| Participants | 8a | Specify the number of individual case safety reports included at each stage, including reasons for exclusion. | yes | *"We extracted 10,832 deduplicated reports and retained 3,389 reports of pregnant individuals exposed to anticancer drugs for the final analysis (Figure 1) (TKI exposure, n=969; other anticancer drugs, n=2,420)."* |
|  | 8b | Provide key demographic and clinical characteristics of cases, if possible comparing cases with any appropriate  reference group. | yes | *"In the group exposed to TKI, most reports were from Asia (n=402, 41.5%), and the mean(SD) age was 28.9(9.5) years old. Within the TKI group, chronic myeloid leukemia (n=724, 92.2%) was the most frequent cancer and other malignancies included acute leukemia (n=24, 3.1%), non-otherwise specified/other leukemia (n=21, 2.7%) and sarcoma (n=16, 2.0%). In the non-exposed group, breast cancer (n=918, 44.3%), other solid tumors (n=409, 19.7%) and lymphoma (n=377, 18.2%) were the most frequent types of cancer (Table1; Figure S2)."* |
| Disproportionality analysis | 9 | Present all results including confidence intervals. Present also results of sensitivity analyses, if performed. | yes | "Pregnancy or fetal/newborn adverse outcomes were identified in 389 reports (40.1%) in the TKI group and 1,524 reports (63.0%) in the other anticancers group, ROR=0.39[95%CI=0.34-0.46] (Figure 2, Table S7)."  "The ROR was significantly higher than 1 in TKI–exposed reports for hydrops fetalis (ROR=13[95%CI=1.5-110], p=0.009), polyhydramnios (ROR=5[95%CI=1.3-20], p=0.02), abortion (ROR=1.8[95%CI=1.4-2.2], p=6x10-7) and threatened preterm labor (ROR=10 [95%CI=1.1-90], p=0.026) (Figure 2)." "In the sensitivity analysis performed on the subpopulation of patients receiving only anticancer in monotherapy (n=1,697, with 896 receiving TKI in monotherapy and 801 other anticancers) trends toward increased risk of polyhydramnios, hydrops fetalis and threatened preterm labor remained (Figure S4&S7)."  "Sensitivity analysis performed on the subpopulation of CML by types of TKI molecules identified similar adverse drug reaction (ADR) outcomes among patients receiving dasatinib with a significant overreporting of hydrops (ROR=8.7 [1.4-53]) and polyhydramnios (ROR=8.7 [1.4-53]) (Figure S8)." |
| Case-by-case analysis | 10 | Present the case-by-case analysis of key variables. Present the causality assessment, if applicable. | No | *No causality assessment* |
| Discussion |  |  |  |  |
| Key results | 11 | Discuss key results with reference to study objectives and contextualize them within the current literature  and other consulted sources. Clearly discriminate between expected reactions and emerging safety  signals. | yes | *"These results are consistent with those previously reported by Pye et al., with 50% of normal infants born after imatinib exposure during pregnancy among 125 pregnancies with known outcomes. In total, 9.6% resulted in infants with fetal abnormalities, including skeletal malformations, renal, respiratory and gastrointestinal abnormalities. In the general population major birth defect risk is around 2 to 3%. In our cohort of declared exposed pregnancies to TKI, we observed a proportion of 5.2% congenital malformations and 3.8% neonatal complications; fetal abnormalities occurred at similar rates than in literature about pregnancy on a TKI, although we cannot make direct comparisons between these pharmacovigilance data and true incidence rate."* |
| External validity | 12a | Discuss the external validity of the results to the general population. | yes | "These results are consistent with those previously reported by […] In addition, since the risk of teratogenicity is well known to date, patients and physicians have adapted their behavior since the first publications.” |
|  | 12b | Discuss the potential relevance of results in clinical practice | yes | " Dasatinib is a dual BCR::ABL1/Src kinase inhibitor, crossing the placenta and leading to considerable levels in fetal plasma […] These results can justify avoiding its use during pregnancy." |
|  | 12c | Propose further study designs if applicable | No | Not applicable |
| Limitations | 13 | Present general limitations, making clear that disproportionality analysis alone cannot prove causation  or measure incidence, and specific limitations, including confounding and reporting bias and efforts to mitigate them. | yes | "This study has some limitations mainly linked to the disproportionality pharmacovigilance approach with inconsistencies in reporting and collection of information. The trimester and duration of exposure to TKIs during pregnancy are major issues in the occurrence of fetal and obstetric complications, which are unfortunately not accurately assessed for each case reported in the database. This could limit the conclusions being drawn about the incidence of these events in the general population. However, apart from abortions, sensitivity analyses carried out among more homogenous subgroups consistently reaffirmed our primary findings."  "Disproportionality analysis alone cannot prove causation or measure incidence. The results are based on reported cases, which are subject to confounding factors such as underreporting or overreporting, and reporting bias, such as the selective reporting of severe or novel adverse events. Efforts to mitigate these biases included sensitivity analyses and subgroup analyses to try to control for confounding and assess the robustness of the findings." |
| Declarations |  |  |  |  |
|  | 14a | Provide the source of funding/sponsorship and the role of the funders/sponsors for the present study and for any original study on which the present article is based. | yes | *"PG was funded by the academic program 'Contrats ED: Programme blanc Institut Curie PSL' during this study. The Institut Curie RT2L research group (PG, ASH, FJ, ED, RK, FC, EL, FR) was supported by the academic program 'SHS INCa', and is part of a research project on young women funded by Monoprix."* |
|  | 14b | Clearly identify potential commercial and intellectual conflicts of interest (e.g., link to any drug/event  investigated, whether financial, legal action, or software used). | yes | *No potential commercial and intellectual conflicts of interest.* |
|  | 14c | Declare any institutional approval needed or granted in the investigation. | yes | *« The institutional review board of Institut Curie (Comité de Revue Institutionnelle-CRI Data) granted study approval. »* |
|  | 14d | Include a statement on data availability, code availability (including the version of the statistical software  used), and protocol registration. | yes | *“The data used in this study are available upon request from the corresponding author. The statistical analyses were performed using RStudio Version 2023.12.1+402.”* |

­

## Table S7. The Strengthening the Reporting of Observational Studies in Epidemiology (STROBE) checklist: guidelines for reporting observational studies.

|  | Item No | Recommendation | Presence in manuscrit | Explication |
| --- | --- | --- | --- | --- |
| Title and abstract | 1 | (a) Indicate the study's design with a commonly used term in the Title or the abstract | yes | Abstract : “this disproportionality analysis study” |
|  |  | (b) Provide in the abstract an informative and balanced summary of what was done and what was found | yes | “All reports with a pregnancy, an antineoplastic treatment during pregnancy, and a cancer were retained. The exposure group was defined as reports mentioning BCR-ABL-TKI.”  “Compared to other anticancer drugs, outcomes overreported with TKIs were hydrops fetalis, polyhydramnios and threatened preterm labor”. |
| Introduction |  |  |  |  |
| Background/rationale | 2 | Explain the scientific background and rationale for the investigation being reported | yes | “Since the early 2000s, the introduction of BCR-ABL tyrosine kinase inhibitors (TKIs) has dramatically improved the outcome of patients with CML and are now the cornerstone of CML management.”  “pregnancy have emerged as an important aspect to be considered in patients of childbearing potential.” |
| Objectives | 3 | State specific objectives, including any prespecified hypotheses | yes | “The main objective of this study was to perform a disproportionality analysis of pregnancy and fetal or newborn adverse outcomes after exposure to BCR-ABL TKI compared to exposure to other anticancer drugs. Secondary analysis included the investigation of molecule-specific toxicities.” |
| Methods |  |  |  |  |
| Study design | 4 | Present key elements of study design early in the paper | yes | “In this cohort study using pharmacovigilance reports from VigiBase, we performed a case/non-case disproportionality analysis to evaluate the association between maternal and fetal/newborn adverse outcomes and exposure to TKI compared to exposure to other anticancer agents” |
| Setting | 5 | Describe the setting, locations, and relevant dates, including periods of recruitment, exposure, follow-up, and data collection | yes | Detailed of population of the study and groups of exposure have been provided. |
| Participants | 6 | (a) Give the eligibility criteria, and the sources and methods of case ascertainment and control selection. Give the rationale for the choice of cases and controls | yes | We have a paragraph on the definition of exposure groups and cases/non cases. |
|  |  | (b) For matched studies, give matching criteria and the number of controls per case | not applicable |  |
| Variables | 7 | Clearly define all outcomes, exposures, predictors, potential confounders, and effect modifiers. Give diagnostic criteria, if applicable | yes | “In this disproportionality analysis, cases were reports with mention of a maternal and fetal/newborn adverse categorized from MedDRA preferred terms in VigiBase. They constituted individual maternal-fetal adverse outcomes regrouped into seven categories for the purposes of this study: Abortion; Stillbirth/fetal death; Congenital malformation; Pregnancy complication; Preterm birth; Neonatal complication; and Delivery complication.” |
| Data sources/ measurement | 8* | For each variable of interest, give sources of data and details of methods of assessment (measurement). Describe comparability of assessment methods if there is more than one group | yes | Information have been provided in “Data query and report extraction” section |
| Bias | 9 | Describe any efforts to address potential sources of bias | yes | We provided extensive analysis of potential biases and confounders in a dedicated section. We also did a multivariate analysis adjusting on relevant confounders. |
| Study size | 10 | Explain how the study size was arrived at | yes | A flowchart has been provided in addition to the explication in the methods section |
| Quantitative variables | 11 | Explain how quantitative variables were handled in the analyses. If applicable, describe which groupings were chosen and why | yes | “The study population is described in terms of frequencies for qualitative variables or medians and interquartile range [IQR] for quantitative variables. Associations between categorical variables were assessed in chi-squared (χ2) tests. P-values of less than 0.05 were considered statistically significant.” |
| Statistical methods | 12 | (a) Describe all statistical methods, including those used to control for confounding | yes | All descriptions are provided in the “Statistical analysis” paragraph. |
|  |  | (b) Describe any methods used to examine subgroups and interactions | yes | Details are in “mitigation of biases and confounding factors” section |
|  |  | (c) Explain how missing data were addressed | yes | “Missing data were grouped within a single level of value for each variable.” |
|  |  | (d) If applicable, explain how matching of cases and controls was addressed | not applicable |  |
|  |  | (e) Describe any sensitivity analyses | yes | Details are in “mitigation of biases and confounding factors” section |
| Results |  |  |  |  |
| Participants | 13* | (a) Report numbers of individuals at each stage of study—eg numbers potentially eligible, examined for eligibility, confirmed eligible, included in the study, completing follow-up, and analysed | yes | “We extracted 10,832 deduplicated reports and retained 3,479 reports of pregnant individuals exposed to anticancer drugs for the final analysis (Figure 1) (TKI exposure, n=991; other anticancer drugs, n=2,488).” |
|  |  | (b) Give reasons for non-participation at each stage | yes | All details are showed in Flowchart with the number of patients at each stage |
|  |  | (c) Consider use of a flow diagram | yes | We provided a flow diagram |
| Descriptive data | 14* | (a) Give characteristics of study participants (eg demographic, clinical, social) and information on exposures and potential confounders | yes | We developed the description of the characteristics of the study population, exposure groups, and potential confounders. |
|  |  | (b) Indicate number of participants with missing data for each variable of interest | yes | Missing values can be seen in Table 1. |
| Outcome data | 15* | Report numbers in each exposure category, or summary measures of exposure | yes | The report numbers are provided for each exposure category. |
| Main results | 16 | (a) Give unadjusted estimates and, if applicable, confounder-adjusted estimates and their precision (eg, 95% confidence interval). Make clear which confounders were adjusted for and why they were included | yes | “In the multivariate analysis (Figure S6), after adjustment for the year of first report in Vigibase and country of the reports, individual’s age, and tumor type …” |
|  |  | (b) Report category boundaries when continuous variables were categorized | yes | We respected this recommendation. |
|  |  | (c) If relevant, consider translating estimates of relative risk into absolute risk for a meaningful time period | not applicable |  |
| Other analyses | 17 | Report other analyses done—eg analyses of subgroups and interactions, and sensitivity analyses | yes | We presented the results of sensitivity/subgroup analyses |
| Discussion |  |  |  |  |
| Key results | 18 | Summarise key results with reference to study objectives | yes | “In this study, we analyzed to our knowledge, the largest cohort of cases of maternofetal exposure to TKI targeting BCR-ABL1 during pregnancy. We found that several TKI-specific adverse outcomes, mainly hydrops fetalis, polyhydramnios and threatened preterm labor, were more frequently reported for TKI treatments than for other anticancer drugs.” |
| Limitations | 19 | Discuss limitations of the study, taking into account sources of potential bias or imprecision. Discuss both direction and magnitude of any potential bias | yes | “This study has some limitations mainly linked to the disproportionality pharmacovigilance approach with inconsistencies in reporting and collection of information.^39,40^ This could limit the conclusions being drawn about the incidence of these events in the general population.” |
| Interpretation | 20 | Give a cautious overall interpretation of results considering objectives, limitations, multiplicity of analyses, results from similar studies, and other relevant evidence | yes | “Nevertheless, delaying the mother’s treatment might lead to severe outcomes in some situations, particularly in cases of highly proliferative disease, accelerated phase or blastic crisis. Whenever “watch and wait” attitude is not possible, primarily imatinib and secondarily nilotinib should be considered, associated with particular attention to threatened preterm labor and exomphalos in the first case.” |
| Generalisability | 21 | Discuss the generalisability (external validity) of the study results | yes | We compared our results to the literature in the Discussion. |
| Other information |  |  |  |  |
| Funding | 22 | Give the source of funding and the role of the funders for the present study and, if applicable, for the original study on which the present article is based | yes | This section is included in our manuscript. |

## Table S8. Pregnancy or fetal/newborn adverse outcomes in the TKI group and in the other anticancer group.

Reporting odds Ratio (ROR) of pregnancy and/or fetal/newborn adverse outcomes in the TKI group compared to the other anticancer group

Abbreviations: CI: Confidence Interval; HT: hypertension; NOS: not otherwise specified; TKI: Tyrosine Kinase Inhibitor; ROR: Reporting odds Ratio

| **category** | **pregnancy or  fetal/newborn outcomes** | **exposed -  BCR-ABL TKI (n=969)** | **non-exposed - other anticancer drugs (n=2420)** | **ROR [95%CI]** | **p-value** |
| --- | --- | --- | --- | --- | --- |
| any adverse outcome |  | 389 (40.1%) | 1524 (63%) | 0.39 [0.34-0.46] | 1.3E-33 |
| abortion |  | 149 (15.4%) | 224 (9.3%) | 1.8 [1.4-2.2] | 5.7E-07 |
|  | spontaneous abortion | 79 (8.2%) | 137 (5.7%) | 1.5 [1.1-2] | 0.01 |
|  | induced abortion | 72 (7.4%) | 91 (3.8%) | 2.1 [1.5-2.8] | 1.6E-05 |
| stillbirth |  | 31 (3.2%) | 112 (4.6%) | 0.68 [0.45-1] | 0.072 |
| congenital malformation |  | 50 (5.2%) | 187 (7.7%) | 0.65 [0.47-0.9] | 0.0073 |
|  | cardiovascular malformation | 17 (1.8%) | 69 (2.9%) | 0.61 [0.36-1] | 0.07 |
|  | neurological malformation | 7 (0.7%) | 18 (0.7%) | 0.97 [0.4-2.3] | 1 |
|  | digestive malformation | 6 (0.6%) | 13 (0.5%) | 1.2 [0.44-3] | 0.8 |
|  | musculoskeletal malformation | 6 (0.6%) | 35 (1.4%) | 0.42 [0.18-1] | 0.055 |
|  | fetal malformation NOS | 5 (0.5%) | 24 (1%) | 0.52 [0.2-1.4] | 0.22 |
|  | genitourinary malformation | 5 (0.5%) | 17 (0.7%) | 0.73 [0.27-2] | 0.64 |
|  | face malformation | 4 (0.4%) | 15 (0.6%) | 0.66 [0.22-2] | 0.61 |
|  | congenital respiratory tract disorder | 4 (0.4%) | 7 (0.3%) | 1.4 [0.42-4.9] | 0.52 |
|  | sensory defect | 3 (0.3%) | 23 (1%) | 0.32 [0.097-1.1] | 0.078 |
|  | genetic disorder | 2 (0.2%) | 11 (0.5%) | 0.45 [0.1-2] | 0.37 |
|  | neurodevelopmental impairment | 1 (0.1%) | 12 (0.5%) | 0.21 [0.027-1.6] | 0.13 |
|  | skin and adnexa anomaly | 1 (0.1%) | 1 (0%) | 2.5 [0.16-40] | 0.49 |
| pregnancy complication |  | 85 (8.8%) | 529 (21.9%) | 0.34 [0.27-0.44] | 4.3E-21 |
|  | intrauterine growth restriction (IUGR) | 30 (3.1%) | 303 (12.5%) | 0.22 [0.15-0.33] | 7.1E-20 |
|  | gestational HT and pre-eclampsia | 15 (1.5%) | 70 (2.9%) | 0.53 [0.3-0.93] | 0.028 |
|  | oligohydramnios | 7 (0.7%) | 134 (5.5%) | 0.12 [0.058-0.27] | 6.1E-13 |
|  | polyhydramnios | 6 (0.6%) | 3 (0.1%) | 5 [1.3-20] | 0.02 |
|  | hydrops fetalis | 5 (0.5%) | 1 (0%) | 13 [1.5-110] | 0.0087 |
|  | threatened preterm labor | 4 (0.4%) | 1 (0%) | 10 [1.1-90] | 0.026 |
|  | gestational diabetes | 3 (0.3%) | 10 (0.4%) | 0.75 [0.21-2.7] | 0.77 |
|  | chorioamnionitis | 3 (0.3%) | 6 (0.2%) | 1.2 [0.31-5] | 0.72 |
|  | pregnancy hemorrhage | 3 (0.3%) | 6 (0.2%) | 1.2 [0.31-5] | 0.72 |
|  | eclampsia | 2 (0.2%) | 1 (0%) | 5 [0.45-55] | 0.2 |
| preterm birth |  | 120 (12.4%) | 709 (29.3%) | 0.34 [0.28-0.42] | 3E-27 |
| neonatal complication |  | 37 (3.8%) | 385 (15.9%) | 0.21 [0.15-0.3] | 5.7E-26 |
|  | neonatal respiratory disorder | 12 (1.2%) | 158 (6.5%) | 0.18 [0.099-0.32] | 1.5E-12 |
|  | neonatal infection | 8 (0.8%) | 56 (2.3%) | 0.35 [0.17-0.74] | 0.0031 |
|  | neonatal hematological disorder | 5 (0.5%) | 109 (4.5%) | 0.11 [0.045-0.27] | 2.8E-11 |
|  | neonatal neuronal disorder | 5 (0.5%) | 43 (1.8%) | 0.29 [0.11-0.73] | 0.0035 |
|  | hyperbilirubinemia | 5 (0.5%) | 39 (1.6%) | 0.32 [0.12-0.81] | 0.011 |
|  | neonatal effusion | 5 (0.5%) | 7 (0.3%) | 1.8 [0.57-5.6] | 0.34 |
|  | neonatal digestive disorder | 4 (0.4%) | 17 (0.7%) | 0.59 [0.2-1.7] | 0.47 |
|  | neonatal metabolic - endocrine disorder | 3 (0.3%) | 27 (1.1%) | 0.28 [0.083-0.91] | 0.024 |
|  | neonatal coagulation disorder | 3 (0.3%) | 7 (0.3%) | 1.1 [0.28-4.1] | 1 |
|  | neonatal renal failure | 1 (0.1%) | 29 (1.2%) | 0.085 [0.012-0.63] | 0.00082 |
|  | neonatal cardiovascular disorder | 1 (0.1%) | 17 (0.7%) | 0.15 [0.019-1.1] | 0.034 |
|  | neonatal skin or adnexa disorder | 1 (0.1%) | 13 (0.5%) | 0.19 [0.025-1.5] | 0.082 |
|  | benign tumor | 1 (0.1%) | 8 (0.3%) | 0.31 [0.039-2.5] | 0.46 |
| delivery complication |  | 12 (1.2%) | 41 (1.7%) | 0.73 [0.38-1.4] | 0.36 |

## Table S9. Subgroups analysis by types of TKI molecules

Reporting odds Ratio (ROR) of pregnancy and/or fetal/newborn adverse outcomes in subgroups analysis by types of TKI molecules

Abbreviations: CI: Confidence Interval; HT: hypertension; NOS: not otherwise specified; TKI: Tyrosine Kinase Inhibitor; ROR: Reporting odds Ratio

|  |  |  | **Imatinib** |  |  |  | **nilotinib** |  |  |  | **dasatinib** |  |  |  | **ponatinib** |  |  |  |
| --- | --- | --- | --- | --- | --- | --- | --- | --- | --- | --- | --- | --- | --- | --- | --- | --- | --- | --- |
| **category** | **ADR** | **n=642** | **ROR [95%CI]** | **p-value** |  | **n=218** | **ROR**  **[95%CI]** | **p-value** |  | **n=127** | **ROR**  **[95%CI]** | **p-value** |  | **n=10** | **ROR**  **[95%CI]** | **p-value** | **bosutinib n=4** | **radotinib n=2** |
| **any adverse outcome** |  | 274 (42.7%) | 0.5 [0.42-0.6] | 7.7E-15 |  | 74 (33.9%) | 0.37  [0.28-0.5] | 6E-12 |  | 41 (32.3%) | 0.35  [0.24-0.52] | 3E-08 |  | 7 (70%) | 1.8  [0.47-7] | 0.53 | 3 (75%) | 1 (50%) |
| **abortion** |  | 103 (16%) | 1.8 [1.4-2.2] | 1.3E-05 |  | 28 (12.8%) | 1.2 [0.8-1.8] | 0.37 |  | 13 (10.2%) | 0.92  [0.51-1.6] | 0.89 |  | 4 (40%) | 5.4  [1.5-19] | 0.018 | 2 (50%) | 0 (0%) |
|  | spontaneous abortion | 53 (8.3%) | 1.4 [1-2] | 0.039 |  | 16 (7.3%) | 1.2 [0.69-2] | 0.57 |  | 7 (5.5%) | 0.85  [0.39-1.8] | 0.85 |  | 2 (20%) | 3.7  [0.78-18] | 0.13 | 1 (25%) | 0 (0%) |
|  | induced abortion | 51 (7.9%) | 2  [1.4-2.9] | 0.00013 |  | 12 (5.5%) | 1.2  [0.64-2.1] | 0.62 |  | 6 (4.7%) | 0.98  [0.43-2.3] | 1 |  | 2 (20%) | 5  [1.1-24] | 0.08 | 2 (50%) | 0 (0%) |
| **stillbirth** |  | 22 (3.4%) | 0.77  [0.48-1.2] | 0.33 |  | 8 (3.7%) | 0.86  [0.41-1.8] | 0.86 |  | 2 (1.6%) | 0.35 [0.087-1.4] | 0.17 |  | 0 (0%) | 0  [0-18] | 1 | 0 (0%) | 0 (0%) |
| **congenital malformation** |  | 37 (5.8%) | 0.78  [0.54-1.1] | 0.2 |  | 10 (4.6%) | 0.62  [0.33-1.2] | 0.17 |  | 2 (1.6%) | 0.21  [0.05-0.84] | 0.011 |  | 1 (10%) | 1.5  [0.19-12] | 0.52 | 0 (0%) | 0 (0%) |
|  | cardiovascular malformation | 9 (1.4%) | 0.49  [0.25-0.99] | 0.05 |  | 6 (2.8%) | 1.1  [0.47-2.5] | 0.82 |  | 2 (1.6%) | 0.61  [0.15-2.5] | 0.77 |  | 0 (0%) | 0  [0-31] | 1 | 0 (0%) | 0 (0%) |
|  | neurological malformation | 7 (1.1%) | 1.7  [0.7-4] | 0.3 |  | 0 (0%) | 0 [0-4.7] | 0.4 |  | 0 (0%) | 0 [0-8.2] | 1 |  | 0 (0%) | 0  [0-NA] | 1 | 0 (0%) | 0 (0%) |
|  | musculoskeletal malformation | 5 (0.8%) | 0.59  [0.23-1.5] | 0.32 |  | 1 (0.5%) | 0.36  [0.049-2.6] | 0.52 |  | 0 (0%) | 0 [0-5] | 0.4 |  | 0 (0%) | 0  [0-66] | 1 | 0 (0%) | 0 (0%) |
|  | digestive malformation | 4 (0.6%) | 1.1  [0.38-3.5] | 0.77 |  | 1 (0.5%) | 0.81  [0.11-6.1] | 1 |  | 0 (0%) | 0 [0-11] | 1 |  | 1 (10%) | 21  [2.5-172] | 0.055 | 0 (0%) | 0 (0%) |
|  | fetal malformation NOS | 4 (0.6%) | 0.68  [0.24-2] | 0.64 |  | 1 (0.5%) | 0.52  [0.07-3.8] | 1 |  | 0 (0%) | 0 [0-7.1] | 0.63 |  | 0 (0%) | 0 [0-94] | 1 | 0 (0%) | 0 (0%) |
|  | genitourinary malformation | 4 (0.6%) | 0.95  [0.32-2.8] | 1 |  | 1 (0.5%) | 0.69  [0.093-5.2] | 1 |  | 0 (0%) | 0 [0-9.4] | 1 |  | 0 (0%) | 0 [0-NA] | 1 | 0 (0%) | 0 (0%) |
|  | face malformation | 4 (0.6%) | 1.1  [0.38-3.5] | 0.77 |  | 0 (0%) | 0 [0-6.1] | 0.63 |  | 0 (0%) | 0 [0-11] | 1 |  | 0 (0%) | 0 [0-NA] | 1 | 0 (0%) | 0 (0%) |
|  | sensory defect | 3 (0.5%) | 0.56  [0.17-1.9] | 0.45 |  | 0 (0%) | 0 [0-4.5] | 0.41 |  | 0 (0%) | 0 [0-7.9] | 0.62 |  | 0 (0%) | 0 [0-NA] | 1 | 0 (0%) | 0 (0%) |
|  | congenital respiratory tract malformation | 3 (0.5%) | 1.6  [0.43-6.1] | 0.45 |  | 1 (0.5%) | 1.5  [0.19-11] | 0.52 |  | 0 (0%) | 0 [0-19] | 1 |  | 0 (0%) | 0 [0-NA] | 1 | 0 (0%) | 0 (0%) |
|  | genetic disorder | 1 (0.2%) | 0.36  [0.046-2.7] | 0.48 |  | 1 (0.5%) | 1.2  [0.16-9.4] | 0.58 |  | 0 (0%) | 0 [0-16] | 1 |  | 0 (0%) | 0 [0-NA] | 1 | 0 (0%) | 0 (0%) |
|  | neurodevelopmental impairment | 1 (0.2%) | 0.36  [0.046-2.7] | 0.48 |  | 0 (0%) | 0 [0-9] | 1 |  | 0 (0%) | 0 [0-16] | 1 |  | 0 (0%) | 0 [0-NA] | 1 | 0 (0%) | 0 (0%) |
|  | skin and adnexa anomaly | 1 (0.2%) | 4.3  [0.27-69] | 0.34 |  | 0 (0%) | 0 [0-61] | 1 |  | 0 (0%) | 0 [0-NA] | 1 |  | 0 (0%) | 0 [0-NA] | 1 | 0 (0%) | 0 (0%) |
| **pregnancy complication** |  | 56 (8.7%) | 0.37  [0.28-0.5] | 3E-13 |  | 16 (7.3%) | 0.34  [0.2-0.57] | 4E-06 |  | 13 (10.2%) | 0.5  [0.28-0.9] | 0.018 |  | 3 (30%) | 1.9  [0.5-7.5] | 0.4 | 1 (25%) | 1 (50%) |
|  | intrauterine growth restriction (IUGR) | 22 (3.4%) | 0.28  [0.18-0.43] | 3E-11 |  | 2 (0.9%) | 0.079  [0.02-0.32] | 5E-08 |  | 7 (5.5%) | 0.53  [0.24-1.1] | 0.13 |  | 1 (10%) | 1  [0.13-8.1] | 1 | 0 (0%) | 0 (0%) |
|  | gestational HT and pre-eclampsia | 10 (1.6%) | 0.56  [0.29-1.1] | 0.093 |  | 5 (2.3%) | 0.91  [0.36-2.3] | 1 |  | 0 (0%) | 0 [0-2.4] | 0.076 |  | 1 (10%) | 4.4  [0.55-35] | 0.22 | 1 (25%) | 0 (0%) |
|  | oligohydramnios | 4 (0.6%) | 0.12  [0.044-0.32] | 8.1E-09 |  | 3 (1.4%) | 0.31  [0.097-0.97] | 0.033 |  | 0 (0%) | 0 [0-1.4] | 0.0099 |  | 0 (0%) | 0 [0-19] | 1 | 0 (0%) | 0 (0%) |
|  | threatened preterm labor | 4 (0.6%) | 17  [1.9-154] | 0.0054 |  | 0 (0%) | 0 [0-24] | 1 |  | 0 (0%) | 0 [0-42] | 1 |  | 0 (0%) | 0 [0-NA] | 1 | 0 (0%) | 0 (0%) |
|  | chorioamnionitis | 3 (0.5%) | 2.1  [0.53-8.6] | 0.38 |  | 0 (0%) | 0 [0-13] | 1 |  | 0 (0%) | 0 [0-23] | 1 |  | 0 (0%) | 0 [0-NA] | 1 | 0 (0%) | 0 (0%) |
|  | polyhydramnios | 3 (0.5%) | 2.1  [0.53-8.6] | 0.38 |  | 1 (0.5%) | 1.8  [0.23-15] | 0.45 |  | 3 (2.4%) | 13  [3.2-53] | 0.0037 |  | 0 (0%) | 0 [0-NA] | 1 | 0 (0%) | 0 (0%) |
|  | gestational diabetes | 2 (0.3%) | 0.78  [0.17-3.5] | 1 |  | 1 (0.5%) | 1.2  [0.16-9.4] | 0.58 |  | 0 (0%) | 0 [0-16] | 1 |  | 0 (0%) | 0 [0-NA] | 1 | 0 (0%) | 0 (0%) |
|  | pregnancy hemorrhage | 2 (0.3%) | 1.2  [0.25-5.9] | 0.68 |  | 0 (0%) | 0 [0-13] | 1 |  | 1 (0.8%) | 3.2 [0.4-26] | 0.29 |  | 0 (0%) | 0 [0-NA] | 1 | 0 (0%) | 0 (0%) |
|  | eclampsia | 2 (0.3%) | 8.6  [0.78-95] | 0.094 |  | 0 (0%) | 0 [0-40] | 1 |  | 0 (0%) | 0 [0-71] | 1 |  | 0 (0%) | 0 [0-NA] | 1 | 0 (0%) | 0 (0%) |
|  | hydrops fetalis | 1 (0.2%) | 0.86  [0.1-7.3] | 1 |  | 1 (0.5%) | 2.9  [0.34-25] | 0.33 |  | 3 (2.4%) | 26  [5.3-130] | 0.001 |  | 0 (0%) | 0 [0-NA] | 1 | 0 (0%) | 0 (0%) |
| **preterm birth** |  | 83 (12.9%) | 0.4  [0.31-0.51] | 2.4E-15 |  | 24 (11%) | 0.36  [0.24-0.56] | 3E-07 |  | 18 (14.2%) | 0.5  [0.3-0.83] | 0.0059 |  | 0 (0%) | 0 [0-2.5] | 0.13 | 0 (0%) | 0 (0%) |
| **neonatal complication** |  | 23 (3.6%) | 0.22  [0.14-0.34] | 4.5E-17 |  | 9 (4.1%) | 0.29  [0.15-0.56] | 3E-05 |  | 5 (3.9%) | 0.28  [0.11-0.69] | 0.0014 |  | 1 (10%) | 0.78 [0.099-6.2] | 1 | 0 (0%) | 0 (0%) |
|  | neonatal respiratory disorder | 9 (1.4%) | 0.23  [0.12-0.45] | 2E-07 |  | 2 (0.9%) | 0.17  [0.041-0.67] | 0.0019 |  | 1 (0.8%) | 0.15  [0.02-1] | 0.02 |  | 0 (0%) | 0 [0-15] | 1 | 0 (0%) | 0 (0%) |
|  | neonatal infection | 5 (0.8%) | 0.36  [0.14-0.89] | 0.023 |  | 2 (0.9%) | 0.46  [0.11-1.9] | 0.44 |  | 1 (0.8%) | 0.4  [0.055-2.9] | 0.52 |  | 1 (10%) | 5.8  [0.73-47] | 0.17 | 0 (0%) | 0 (0%) |
|  | hyperbilirubinemia | 5 (0.8%) | 0.55  [0.21-1.4] | 0.25 |  | 0 (0%) | 0 [0-2.6] | 0.11 |  | 1 (0.8%) | 0.59 [0.081-4.3] | 1 |  | 0 (0%) | 0 [0-62] | 1 | 0 (0%) | 0 (0%) |
|  | neonatal neuronal disorder | 4 (0.6%) | 0.39  [0.14-1.1] | 0.063 |  | 1 (0.5%) | 0.31  [0.042-2.2] | 0.37 |  | 0 (0%) | 0 [0-4.2] | 0.26 |  | 0 (0%) | 0 [0-57] | 1 | 0 (0%) | 0 (0%) |
|  | neonatal hematological disorder | 3 (0.5%) | 0.11  [0.035-0.35] | 1.5E-07 |  | 2 (0.9%) | 0.25  [0.062-1] | 0.032 |  | 1 (0.8%) | 0.22 [0.031-1.6] | 0.13 |  | 0 (0%) | 0 [0-23] | 1 | 0 (0%) | 0 (0%) |
|  | neonatal digestive disorder | 3 (0.5%) | 0.71  [0.21-2.4] | 0.78 |  | 0 (0%) | 0 [0-5.6] | 0.4 |  | 1 (0.8%) | 1.3  [0.17-9.7] | 0.55 |  | 0 (0%) | 0 [0-NA] | 1 | 0 (0%) | 0 (0%) |
|  | neonatal effusion | 3 (0.5%) | 1.4  [0.39-5.3] | 0.71 |  | 0 (0%) | 0 [0-9.8] | 1 |  | 2 (1.6%) | 5.2 [1.1-24] | 0.072 |  | 0 (0%) | 0 [0-NA] | 1 | 0 (0%) | 0 (0%) |
|  | neonatal coagulation disorder | 3 (0.5%) | 1.8  [0.47-7.1] | 0.41 |  | 0 (0%) | 0 [0-12] | 1 |  | 0 (0%) | 0 [0-21] | 1 |  | 0 (0%) | 0 [0-NA] | 1 | 0 (0%) | 0 (0%) |
|  | neonatal metabolic - endocrine disorder | 2 (0.3%) | 0.3  [0.072-1.3] | 0.1 |  | 0 (0%) | 0 [0-3.9] | 0.26 |  | 1 (0.8%) | 0.88 [0.12-6.5] | 1 |  | 0 (0%) | 0 [0-91] | 1 | 0 (0%) | 0 (0%) |
|  | neonatal cardiovascular disorder | 1 (0.2%) | 0.25  [0.033-1.9] | 0.23 |  | 0 (0%) | 0 [0-6.5] | 0.63 |  | 1 (0.8%) | 1.5 [0.2-11] | 0.5 |  | 0 (0%) | 0 [0-NA] | 1 | 0 (0%) | 0 (0%) |
|  | neonatal skin or adnexa disorder | 1 (0.2%) | 0.33  [0.043-2.5] | 0.49 |  | 0 (0%) | 0 [0-8.4] | 1 |  | 0 (0%) | 0 [0-15] | 1 |  | 0 (0%) | 0 [0-NA] | 1 | 0 (0%) | 0 (0%) |
|  | neonatal renal failure | 0 (0%) | 0  [0-1.1] | 0.0036 |  | 1 (0.5%) | 0.5  [0.068-3.7] | 0.72 |  | 0 (0%) | 0 [0-6.8] | 0.63 |  | 0 (0%) | 0 [0-91] | 1 | 0 (0%) | 0 (0%) |
|  | benign tumor | 0 (0%) | 0  [0-3.9] | 0.22 |  | 1 (0.5%) | 1.8  [0.23-15] | 0.45 |  | 0 (0%) | 0 [0-23] | 1 |  | 0 (0%) | 0 [0-NA] | 1 | 0 (0%) | 0 (0%) |
| **delivery complication** |  | 9 (1.4%) | 0.87  [0.42-1.8] | 0.86 |  | 3 (1.4%) | 0.87  [0.27-2.8] | 1 |  | 0 (0%) | 0 [0-3.8] | 0.27 |  | 0 (0%) | 0 [0-51] | 1 | 0 (0%) | 0 (0%) |

# Supplemental Methods

## Identification of reports addressing mother vs fetal/newborn

### Position of the problem

To better assess adverse outcomes. we attributed each report to the mother or the fetus/child. Attribution was made using a scoring system.

### Scoring system for each report

| **Variable** | **Modality** | **Mother score** | **Fetal/newborn score** |
| --- | --- | --- | --- |
| Age group | Neonate. child, infant |  | 2 |
|  | Adult | 2 |  |
| Age unit | Days, day, months, month, hour, hours, weeks |  | 2 |
| Age | <10 |  | 1 |
| Age unit  Age | Age unit = year  & Age >10 | 2 |  |
| Weight | <10 |  | 1 |
|  | >10 | 1 |  |
| Sex | Male |  | 1 |
| Reports seriousness criteria | Congenital anomaly/birth defect |  |  |
| Drug route administration | Transplacental |  | 2 |
| Reaction PT | "Drug exposure in utero",  "Foetal exposure during pregnancy",  "Foetal exposure during delivery",  "Foetal exposure timing unspecified" |  | 1 |
| Reaction PT | "Drug exposure before pregnancy",  "Maternal exposure before pregnancy",  "Exposure during pregnancy",  "First trimester pregnancy",  "High risk pregnancy",  "Maternal exposure during delivery",  "Maternal exposure during pregnancy",  "Pregnancy",  "Pregnant",  "Pregnancy on contraceptive",  "Pregnancy on oral contraceptive",  "Pregnancy with advanced maternal age",  "Pregnancy with contraceptive device",  "Pregnancy with injectable contraceptive",  "Unintended pregnancy",  "Unwanted pregnancy",  "Maternal exposure timing unspecified" | 1 |  |
| Reaction PT | PT, HLT or HLGT code for a malignant tumor (SOC = "Neoplasms benign, malignant and unspecified (incl cysts and polyps)", terms include lymphoma, leukemia, myeloma, malignant, malignancy, sarcoma, carcinoma, “metast” | 1 |  |


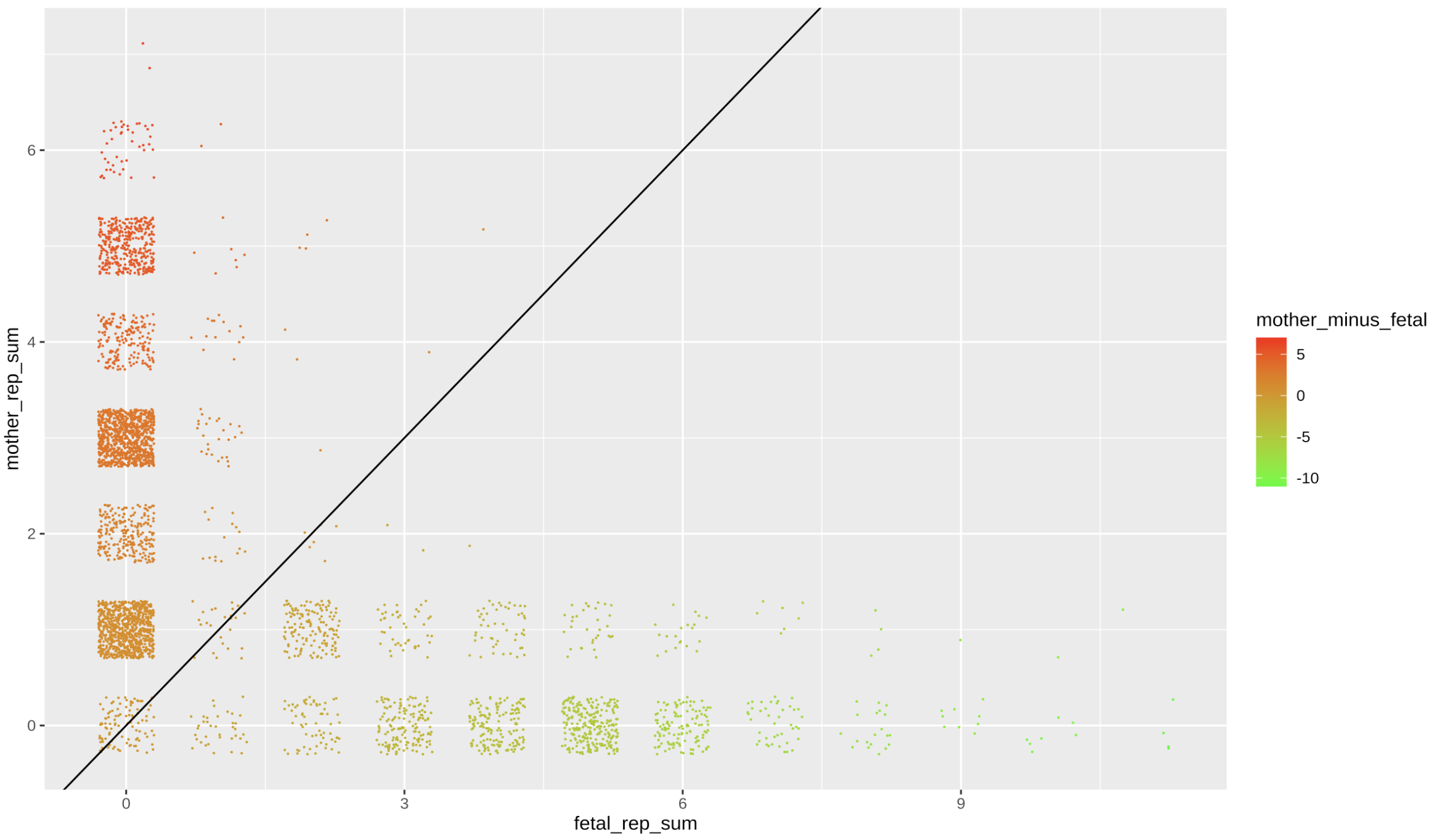


Figure: Difference of the score for maternal items (mother_rep_score) with fetal/newborn items (fetal_rep_sum)

## Final attribution

Reports with “score mother” > “score fetal/newborn” were attributed to the mother and reports with “score mother” < “score fetal/newborn” were attributed to the fetus/newborn.

132 reports on 3977 (3.3%) screened had a “score mother” = “score fetal/newborn” and were deciphered individually.

## Deduplication algorithm

### Problem position

Due to the possibility of reporting from different sources inherent to pharmacovigilance, there is an important possibility than some cases could be reported multiple times in VigiBase. To address this problem, the Upsala Monitoring Center, within the “VigiLyze” platform, integrated a duplication detection algorithm, “VigiMatch”. However, from our experience, and particularly in the VITALITY cohort, there remains a risk that some cases could still be reported multiple times, notably because some reports are also extracted from literature.

To address this problem, we developed an in-house information entropy-based algorithm of detection of duplicates.

### Initial selection

First, we selected all reports for which we found:

1. Same country origin
2. Same anticancer drugs declared within report
3. Report addressing mother or fetus/newborn

Then, all the tandems of potential duplicates were evaluated.

Information entropy

For each variable considered, i.e. country of report, we evaluated the amount of information brought by the report using an entropy-based scoring system.

For each tandem, we calculated an entropy-based score of the similarities of reports.

Entropy score for each variable was calculated with the following formula:

$$\frac{Ntot}{n}. log(\frac{Ntot}{n})$$

Were:

- **Ntot** is the total number of total reports in the cohort
- **n** is the number of occurrences of the variable

For example, a tandem with France as the same country had a “country” entropy score of:

n=151 reports from France and N_tot_ is 3956

S_country_ = 3956/151.log(3956/151) = 85.6

Total entropy score was calculated from the entropy of the following variable:

- country
- age
- weight
- size
- mother age
- mother size
- mother weight
- date of last menstruation
- cancer type
- year of first report
- year of first event reported
- drugs
- start date of drugs
- reported and MedDRA PT-mapped adverse event
- start date of adverse events
- mapped materno-fetal adverse events

for drugs and adverse events, each match was counted individually

Score validation and determination of entropy score threshold using reports which were also reported in the literature and for which the reference was available within the CIOMS extracted file, and for whom the status of duplicate was unambiguous, we found that a threshold of 2000 was had a specificity of 100%.


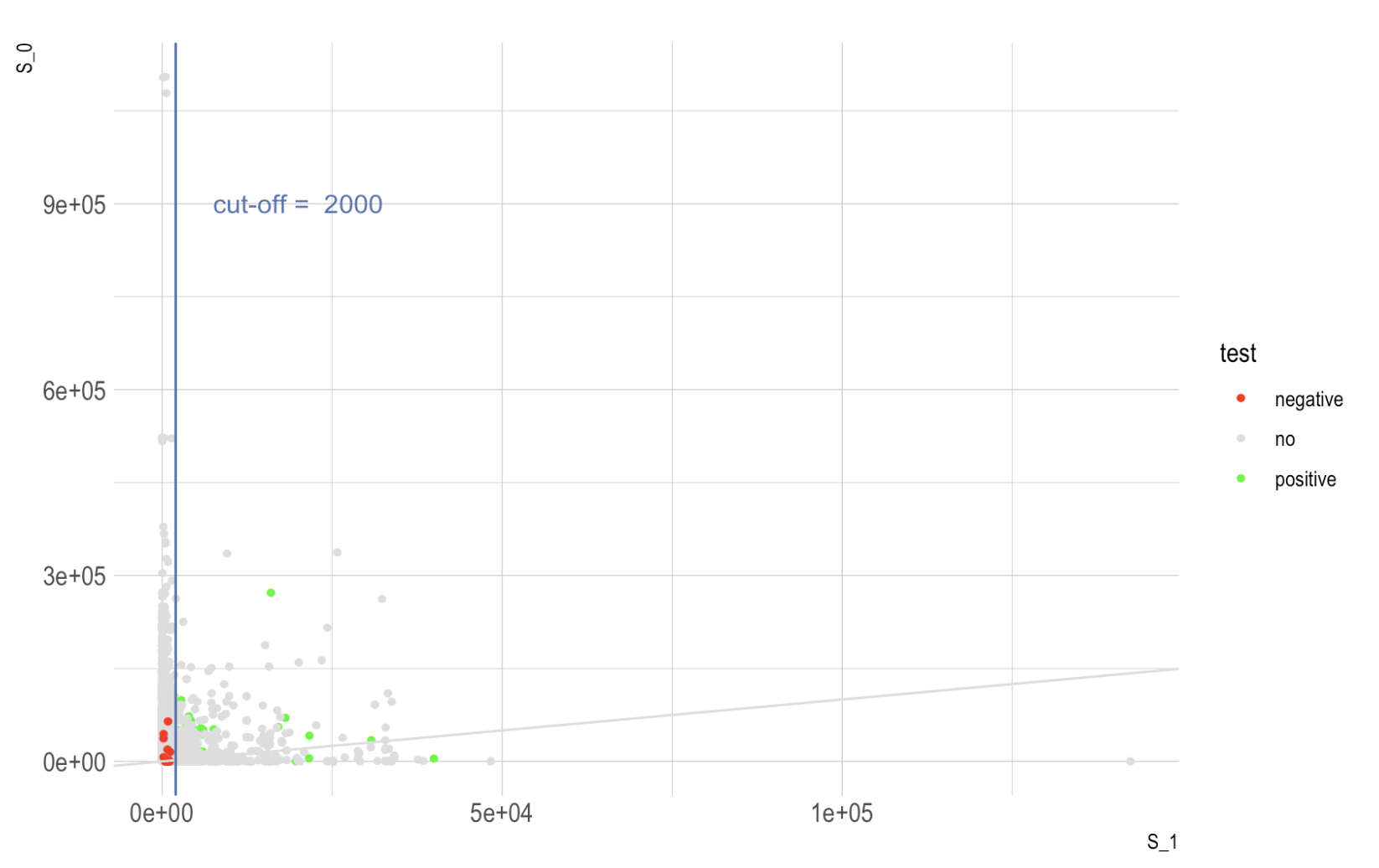


Figure. Entropy score S_1 and score for discordance S_0 for 46,159 tandems of reports from 3,956 reports within the cohort. Test were performed on reports that had a literature reference within the CIOMS extracted case.

Of note, same reports could have a discordance entropy elevated when additional and rarely reports information are present in one of the duplicates and not the other one.

### Fusion of duplicates

After detection of duplicates, information from reports were mixed within a single report using available data for all demographic data and drugs and adverse events. For demographic data, whenever there was a discrepancy, data from the latest reports was kept.

## Fusion of dyads

### Position of the problem

In some situations, for a single case, 2 reports could be declared for both the fetus/newborn and the mother. This present two problems: first, the denominator is modified. Second, for materno-fetal toxicity such as hydrops fetalis, both the mother and the child could be declared individually, amplifying a fake signal.

Initial selection of dyads

First, we selected all reports for which we found:

### Inclusion criteria

1. Same country origin
2. Same anticancer drugs declared within report
3. Same cancer identified in report
4. Date of first declaration with a latitude of 1 year
5. Date of first event with a latitude of 1 year
6. One of the reports is addressing the mother and the second is addressing the fetus/newborn

### Exclusion criteria

- different last menstruation date

- different mother weight size and age

- dyads from published literature not compatible with dyads

Then, all the tandems of potential dyads were evaluated using entropy score.

An entropy score of 500 was deemed relevant. Dyads were merged and interpreted as one single report.
